# Optimising hybrid renewable energy systems for remote tribal villages: A techno-economic case study from central and Eastern India

**DOI:** 10.1038/s41598-026-45306-6

**Published:** 2026-04-01

**Authors:** Y. Raja Sekhar, C. Chiranjeevi, Ravindra, Muhammad Asif, Mohamed Bechir Ben Hamida, Gabr Goshu Syum

**Affiliations:** 1 School of Mechanical Engineering, Vellore Institute of Technology, Vellore, 632014 India; 2 Nitte (Deemed to be university), NMAM Institute of Technology (NMAMIT), Nitte, Department of Mechanical Engineering, Mangalore, 574110 Karnataka India; 3 Department of Mathematics and Statistics, College of Sciences, Imam Mohammad Ibn Saud Islamic University (IMSIU), Riyadh, Saudi Arabia; 4 Deanship of Scientific Research, Imam Mohammad Ibn Saud Islamic University (IMSIU), Riyadh, Saudi Arabia; 5 Faculty of Mechanical and Industrial Engineering, EiT-M, Mekelle University, P. O. Box 231, Mekelle, Tigray, 7000 Ethiopia; 6 Centre of Studies in Oil and Gas Engineering and Technology(CS-OGET), Eduardo Mondlane University, Bairro Luís Cabral, Moçambique, Maputo, Mozambique

**Keywords:** Hybrid Renewable Energy Systems, Solar Photovoltaics, HOMER Software, Net present cost, levelized energy cost, sustainability, Energy and society, Energy science and technology, Engineering, Environmental sciences

## Abstract

Hybrid renewable energy systems (HRES) offer a sustainable and resilient solution for meeting domestic energy demands in remote regions and combating climate change. This study assessed the technical and economic feasibility of establishing a self-sufficient HRES for the remote tribal villages of Koopgarh, Madhya Pradesh, and Kurkheta, Jharkhand, India, as a case study. These regions, owing to their dense forest cover, are not connected to the national grid but offer significant potential for tapping into solar, wind, and micro hydropower. Hence, site-specific HRES configurations were proposed in this study, considering solar PV, Wind, Battery energy storage, and Diesel Generators, along with current grid connectivity. In addition to traditional metrics such as net present cost (NPC), levelized cost of energy (LCOE), and environmental emissions for HRES configurations, a sensitivity analysis considering future variations in resource availability and energy demands was presented to understand the influence of capital cost shortages on LCOE. The proposed hybrid photovoltaic-wind battery (PV-WT-B) system, supplemented with a battery energy storage system for improved efficiency and safety, is the most viable option. Simulation results show that the proposed HRES can achieve an LCOE of USD 0.193 per kilowatt-hour for Koopgarh and USD 0.033 per kilowatt-hour for Kurkheta, while significantly reducing the carbon footprint and promoting energy equity in rural areas. This approach goes beyond mere energy generation and creates a sustainable, self-reliant community that satisfies the current and future energy demands of the region.

## Introduction

Energy shortages in various regions pose a significant barrier to development in many emerging nations, where electricity generation relies heavily on conventional fossil fuels. Recently, growing global concerns about the depletion of these resources and their environmental impact, particularly their role in climate change, have led to increased interest in renewable energy alternatives. These renewable sources are abundant, sustainable, and environmentally sound, offering a cost-efficient solution for electricity access in developing countries^1^. However, solar and wind resources are inherently intermittent, posing challenges in consistently meeting energy demands. For instance, solar energy is only available during daylight, whereas wind turbines reach peak performance only during specific months with ideal wind speeds. Consequently, relying on a single renewable source for continuous power is often unfeasible in these regions. To address this issue, hybrid renewable energy systems (HRES) are employed. These systems combine multiple energy sources, such as solar, wind, and hydro, to ensure a more stable and reliable power supply.

Hybrid Renewable Energy Systems (HRES) also offer greater sustainability, generating electricity without emissions and contributing minimally to air pollution and greenhouse gas output. Moreover, HRES have become more economically viable than traditional power plants, owing to the declining prices of solar panels and wind turbines, making them increasingly accessible. Numerous theoretical studies have been conducted by researchers employing Artificial Neural Networks (ANN) and other optimization algorithms to enhance the performance and efficiency of various energy systems^2–5^. In developing nations, HRES offers a promising solution to the ongoing energy crisis by providing a reliable, eco-friendly, and cost-effective power supply. As the production costs of renewable energy continue to decrease, the adoption of HRES is expected to grow substantially. Beyond their core advantages, HRES provides several added benefits, such as^6–9^:

- Reducing emissions from conventional fossil fuel-based power generation, thereby improving the quality of local air.
- Generating employment opportunities in the renewable energy industry.
- Decreasing reliance on imported fuels, which contributes to cost savings and strengthens energy independence.

Numerous studies have been conducted to determine the most suitable HRES configurations for specific energy requirements. One of the most commonly used simulation tools for assessing and analyzing HRES is the HOMER Pro Microgrid, developed by the National Renewable Energy Laboratory (NREL). For instance, Baruah et al.^10^ investigated the viability of a standalone HRES designed to supply power to a settlement in the East District of Sikkim, India, using the HOMER PRO Microgrid Analysis tool version 3.14.2. Their proposed system incorporates solar power and other renewable sources, complemented by battery storage for backup. Their findings demonstrated that the system was financially feasible, with an average Levelized Cost of Energy (LCOE) of $0.095 per kWh^10^. In another study, Suresh and Kumari^11^ employed an improved genetic algorithm (GA) to optimize the sizing of hybrid energy systems comprising diesel generators, photovoltaic arrays, wind turbines, and battery storage units. This optimization technique successfully minimized the total system cost while ensuring a reliable energy supply and meeting demand.

Kumar et al.^12^ used HOMER PRO Microgrid Analysis tool version 3.13 to optimize a cost-effective, grid-connected microgrid to ensure an uninterrupted power supply in Jalalabad village. Their analysis showed that a 1.2 MW system with an initial cost of INR 20 million had an LCOE of INR 11.23/kWh (USD 0.13) and an NPC of INR 15 million (USD 17,968). Incorporating demand-side strategies reduced both the LCOE and NPC by up to 20%. Similarly, Murugaperumal et al.^13^ assessed an HRES for Korkadu village in Puducherry that integrated PV, wind, diesel, and battery systems using HOMER PRO Microgrid Analysis tool version 3.13. The system cost USD 150,000, with an LCOE of USD 0.18/kWh and an NPC of USD 300,000, offering better economic value than a grid-connected setup. Mokhtar et al.^14^ developed an HRES design for off-grid homes in arid regions using a combination of demand-side management (DSM) and Particle Swarm Optimization (PSO). Their approach reduced the Net Present Cost (NPC) by up to 18% and increased renewable energy (RE) integration from 15% to 63%.

Ramesh and Saini^15^ studied an HRES with PV, wind, and batteries for a rural Indian community and evaluated two DSM methods: strategic conservation and peak shifting. These strategies cut the NPC by 33% and 25%, respectively, and reduced the energy cost by $0.003/kWh and $0.002/kWh. Ali et al.^16^ examined an HRES comprising a PV-WES-battery-inverter system for Gwadar, Pakistan, with an LCOE of $0.0347/kWh, lower than conventional sources. Jeyasudha et al.^17^ assessed a PV-wind-battery-converter system for Indian villages, reporting an LCOE of $0.05/kWh. Saiprasad et al.^18^ used the iHOGA tool to analyze the technical, economic, and social impacts of an HRES in a small Indian town.

Das et al.^19^ optimised a solar-biogas-PHES-battery setup for a radio station using WCA, MFO, and GA. The WCA method yielded the lowest NPC of $0.813 million, with a PV capacity of 69.2 kW and biogas generator capacity of 16 kW. Malik et al.^20^ evaluated seven biomass-based HRES configurations in the Himalayas, concluding that a PV-biomass-energy storage system had the lowest LCOE ($0.185/kWh) and reduced CO₂ emissions by 27.8 million metric tons annually. Kumar and Channi^21^ modelled a PV-biomass HRES for Punjab’s Sidhwanbet region, serving 770 households. The system demonstrated better cost-effectiveness and lower emissions than standalone alternatives. Kushwaha and Bhattacharjee^22^ found predictive dispatch to be the most efficient method in terms of cost, reliability, emissions, and land use.

Chingkheinganba et al.^23^ also favoured predictive dispatch in both grid-tied and standalone HRES setups, noting its resilience to economic and component variability. In Baluchistan, a similar system reduced operating costs and CO₂ by 9% and 957,477 kg, respectively^24^. Aziz et al.^25^ assessed a diesel-PV-battery HRES in Iraq using HOMER PRO Microgrid Analysis tool version 3.14.2 with MATLAB Link. Their proposed system, with 41.3% renewables, had an NPC of $4.03 million and 851,377 kg CO₂/year—outperforming the default cycle charging (33.9% renewables, $4.19 million NPC, 957,477 kg CO₂/year). Lotfi et al.^26–32^ conducted multi-objective optimisation to identify optimal solutions across various renewable energy scenarios. However, most past HRES studies have paid limited attention to load demand variations and regional economic factors, often relying on assumptions that may not align with real-world load fluctuations. Although steady loads are easier to model, rising energy needs driven by local population growth demand more site-specific approaches.

Yadav et al.^33^ studied a hybrid renewable energy system (HRES) with multiple energy storage systems (MESS) that integrated batteries and hydrogen storage. Using HOMER PRO Microgrid Analysis tool version 3.16.2, a techno-economic analysis was conducted on a microgrid comprising wind turbines, solar PV, fuel cells, electrolyzers, HSS, and BES. The goal was to minimise the Net Present Cost (NPC) and the Cost of Energy (COE) across different configurations. The optimal configuration of WT/PV/FC/electrolyser/HSS/BES/converter yielded the lowest NPCs of $838,832 and $679,605, and COEs of $0.232/kWh and $0.189/kWh, respectively, at 0.0% and 1.0% capacity shortages. Sensitivity analysis highlighted the effects of techno-economic uncertainties on the system costs. Hourly data enables 100% renewable coverage at a diesel cost of $0.46/kWh, whereas minute-level data limits coverage to 85.87–95.64% owing to demand and generation volatility. Recently, Qasim et al.^35^ performed techno-economic optimization studies for a region in Iraq, considering various energy sources, including battery energy storage systems. They identified the best combination of energy resources for the lowest LCOE: PV/WT/BESS/DG combination. Furthermore, other tools, such as HyDesign, have been developed to perform grid optimization studies using various renewable energy sources as inputs, along with battery energy storage.

Sustainable rural electrification is a critical requirement for remote and tribal regions, where difficult terrain, sparse population, high transmission losses, and elevated infrastructure costs often constrain conventional grid extension. In many such areas, reliance on diesel generators or intermittent grid supply results in high operational costs, fuel insecurity and increased greenhouse gas emissions. Sustainable electrification through hybrid renewable energy systems (HRES) offers a decentralized, resilient solution by utilizing locally available renewable resources such as solar and wind energy. Beyond providing reliable electricity access, sustainable rural electrification supports socioeconomic development by improving healthcare, education, communication, and livelihood opportunities while simultaneously reducing environmental impacts and enhancing energy equity. Hence, adopting sustainable and site-specific HRES configurations is essential for achieving long-term energy security and sustainable development in remote rural communities in India.

Earlier research has shown that utilizing HOMER software for rural electrification in India’s remote areas is beneficial, but several research gaps and limitations persist. These disparities arise from technical, socioeconomic, geographic, and data-related challenges. Studies on HRES have employed varying assumptions to recommend an optimal configuration of hybrid systems based on the most suitable resources available at a given location. However, these assumptions are not always suitable for real-time load demands, which frequently fluctuate between energy demand and supply. However, replicating steady loads across multiple locations is relatively straightforward. As Version 4previous studies on HRES have demonstrated, it is essential to consider the increasing energy demands caused by the expanding local population. Hence, the present analytical study emphasizes these gaps by selecting appropriate renewable sources tailored to each tribal site, where power access is limited owing to difficult terrain. The novelty of this study is that there is no existing literature on HRES studies for remote locations in the northern tribal regions of India, considering future domestic energy demands. The proposed HRES meets local energy demands, as validated through technical and financial analyses. Among various non-conventional options, this study recommends the most efficient hybrid setups per site based on LCOE calculations that factor in reduced battery energy storage and potential CO₂ emissions.

HOMER PRO Microgrid Analysis tool version 3.16 tool was used to model and optimize various hybrid system configurations, comprising grid, photovoltaic (PV), wind turbine (WT), and battery energy storage systems (BESS), for remote tribal communities in northern India. By maximizing the proportion of renewable energy, this study evaluated optimal hybrid renewable energy system (HRES) configurations based on site-specific meteorological data, identifying solutions with the lowest levelized cost of energy (LCOE) and net present cost (NPC). A comprehensive techno-economic analysis was conducted, comparing the performance of each configuration by assessing the capital, operating, and replacement costs, as well as the fuel consumption and carbon emissions. This analysis contrasts existing grid connections with off-grid alternatives to underscore the economic advantages of HRES deployment. Additionally, this study quantifies the potential reduction in CO₂ emissions from the transition from fossil-fuel-based energy systems to hybrid renewable systems, highlighting the environmental benefits of a high renewable energy fraction, particularly for isolated regions reliant on diesel generators. Furthermore, this study proposes a scalable hybrid power system design that is adaptable to areas with limited or unreliable grid access. The model’s robustness is further demonstrated through sensitivity analyses that account for variations in future energy resource availability, ensuring its reliability in meeting the annual energy demand. Ultimately, the results provide a practical framework for policymakers and energy planners to implement sustainable, cost-effective, and environmentally beneficial hybrid renewable energy systems.

The proposed hybrid renewable energy systems (HRES) directly contribute to several United Nations Sustainable Development Goals (SDGs). This study advances SDG 7 by delivering reliable, affordable, and clean energy to remote communities while reducing diesel dependence and emissions, thereby supporting SDG 13. Decentralized renewables create local jobs (SDG 8) and improve access to healthcare and education (SDGs 3 and 4). Overall, the HRES framework strengthens energy equity, resilience, and sustainable rural development in rural areas.

## Methodology

**Fig. 1:**
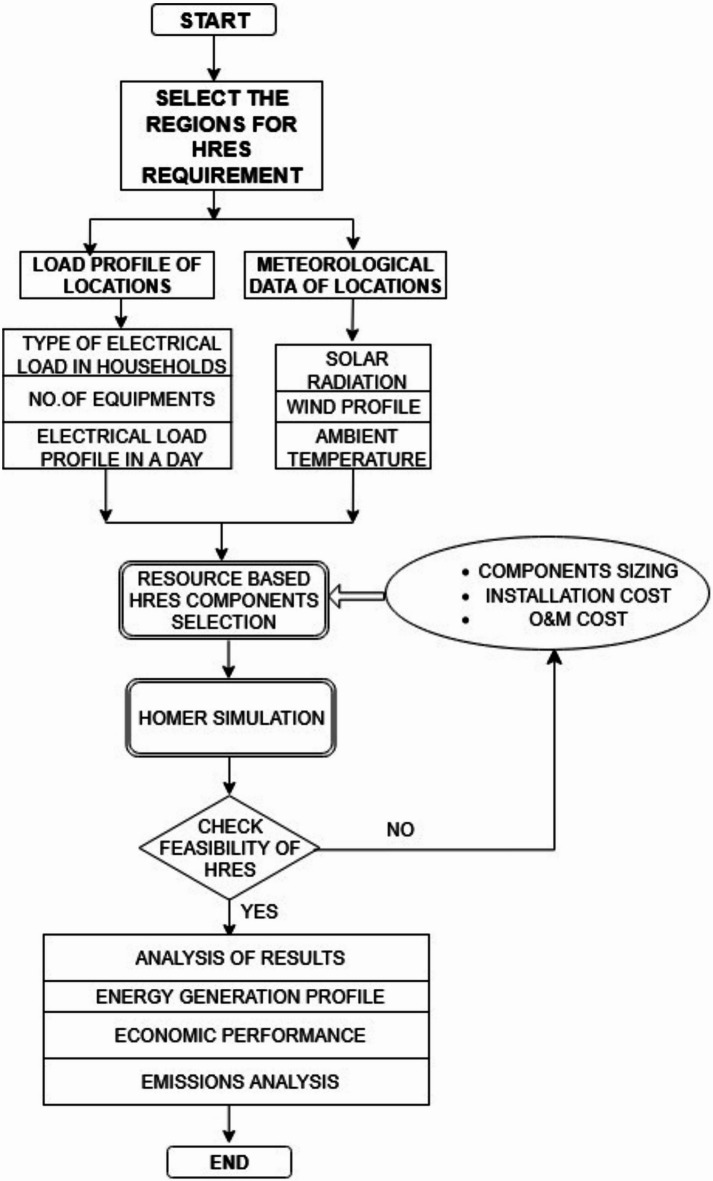
Methodology Flow chart of simulation.

Access to reliable electricity in many rural parts of India remains a significant challenge, with grid connections often proving inconsistent and insufficient to meet the actual energy demands. This study identified two locations where grid connectivity issues were particularly acute. Data on locally available renewable resources were collected, and an assessment was conducted to recommend suitable HRES configurations. The performance of these systems was evaluated through simulations using HOMER software, a tool well-suited for designing both off-grid and grid-connected setups that integrate diverse renewable sources, such as photovoltaic panels, solar thermal units, wind energy generators (WEG), and small-scale hydropower. HOMER also provides built-in load profiles tailored to residential, commercial, and community energy needs, while offering the flexibility to input custom demand data as needed. Based on a comprehensive evaluation, the most appropriate hybrid configurations were selected for the simulations at the respective sites. During these simulations, the software enabled the setting of a buy-back tariff, simulating conditions where surplus power was fed back into the grid. This feature supports reducing upfront system costs, ensures that energy demand is met effectively, minimizes energy wastage, and helps maintain cost efficiency. The methodology adopted in this study is illustrated in Fig. 1. Techno-economic simulations and optimization of the hybrid renewable energy systems were performed using HOMER Pro Microgrid software (Version 3.16, licensed 2023, developed by the National Renewable Energy Laboratory (NREL), USA).

### Site Selection for analysis

**Fig. 2:**
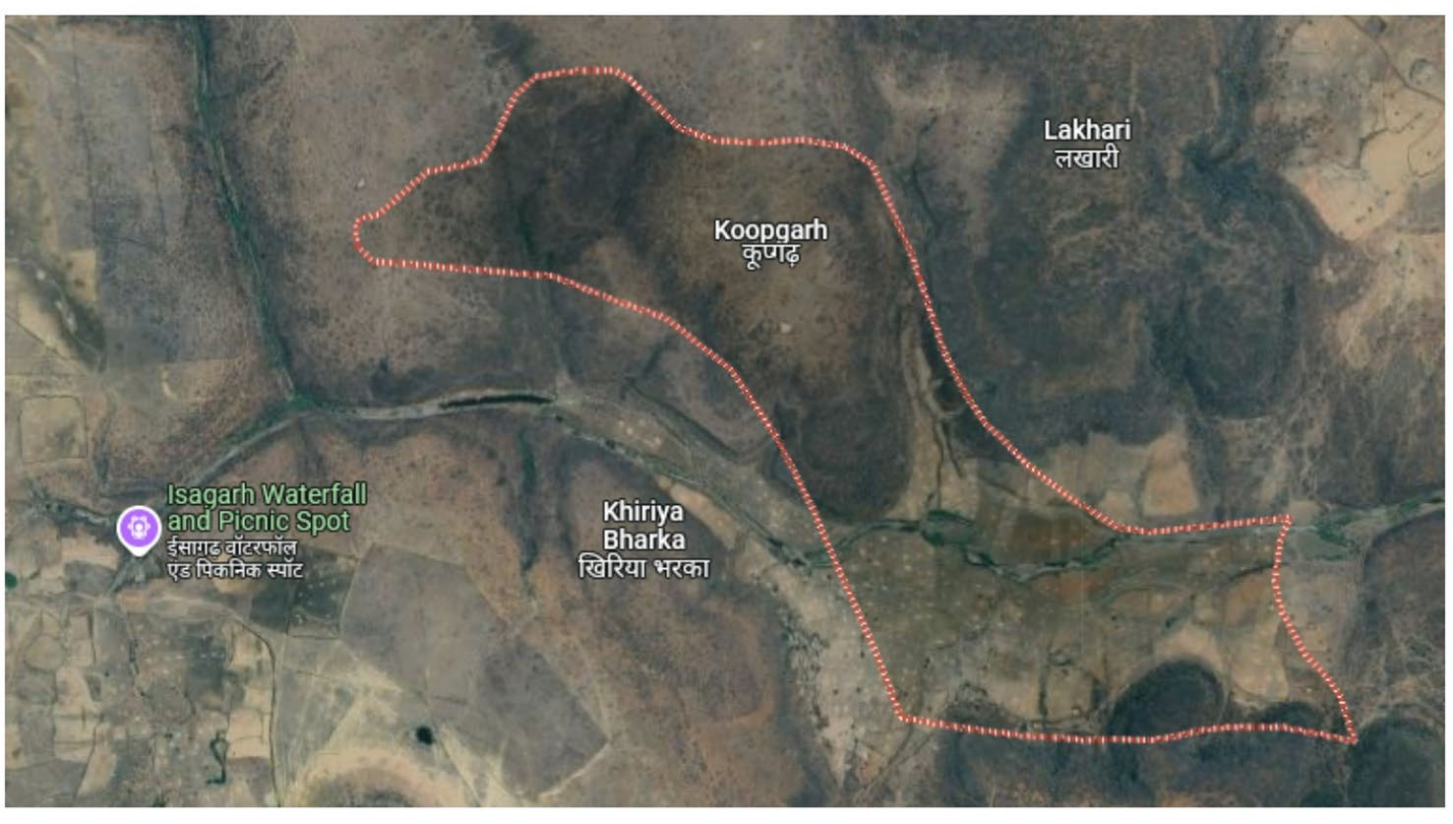
Aerial View Koopgarh village. (Source: Google Maps).

**Fig. 3:**
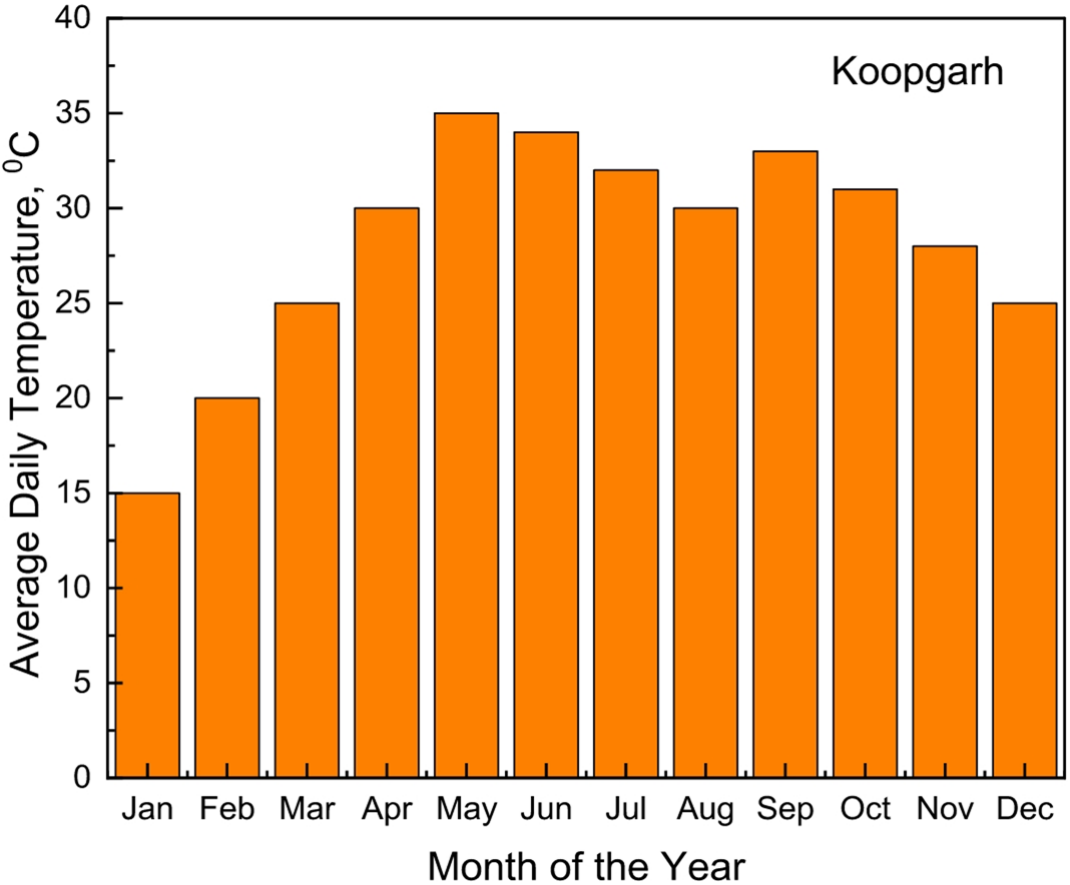
Variation in Monthly Average Daily Temperature at Koopgarh site.

**Fig. 4:**
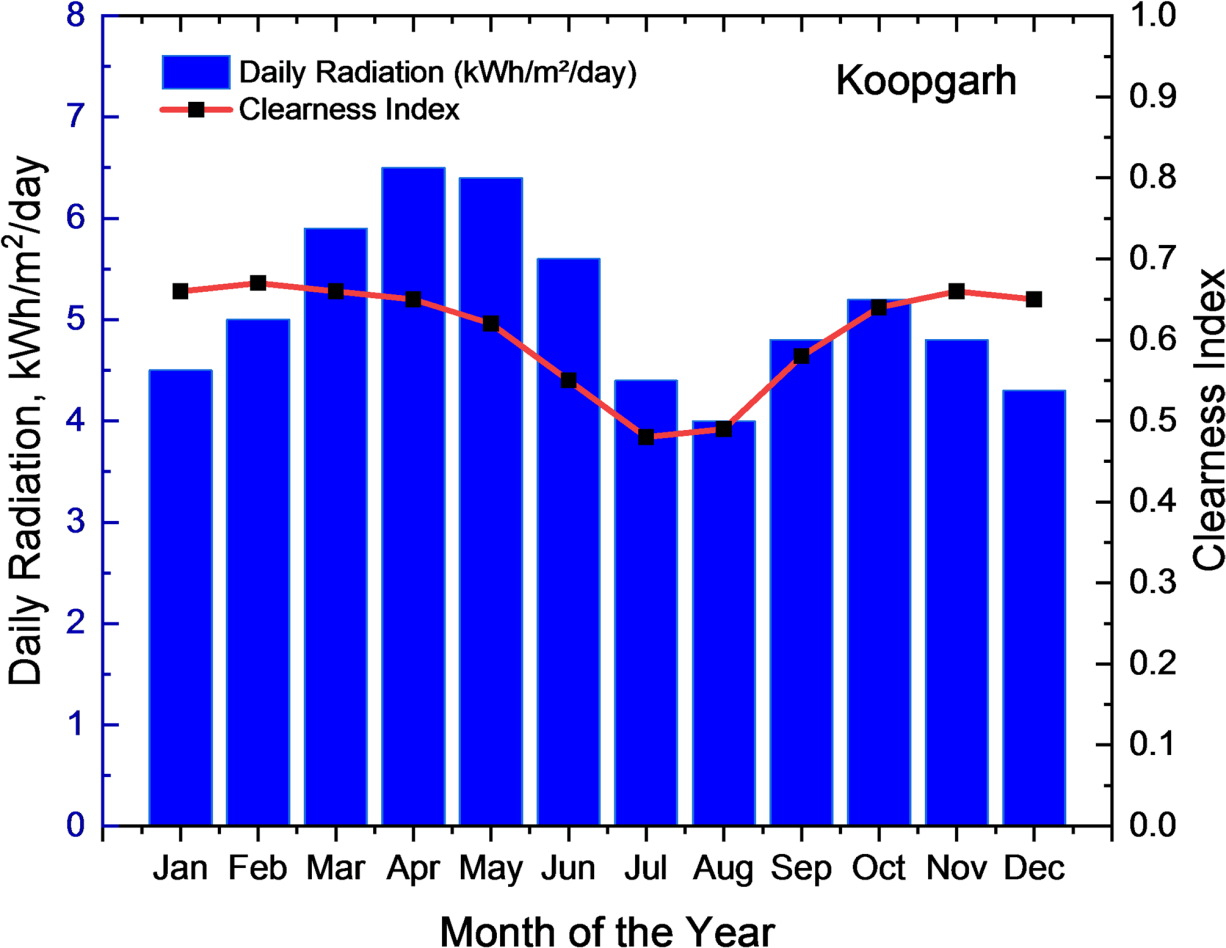
Average Monthly Solar Global Horizontal Irradiance (GHI) Index for Koopgarh.

**Fig. 5:**
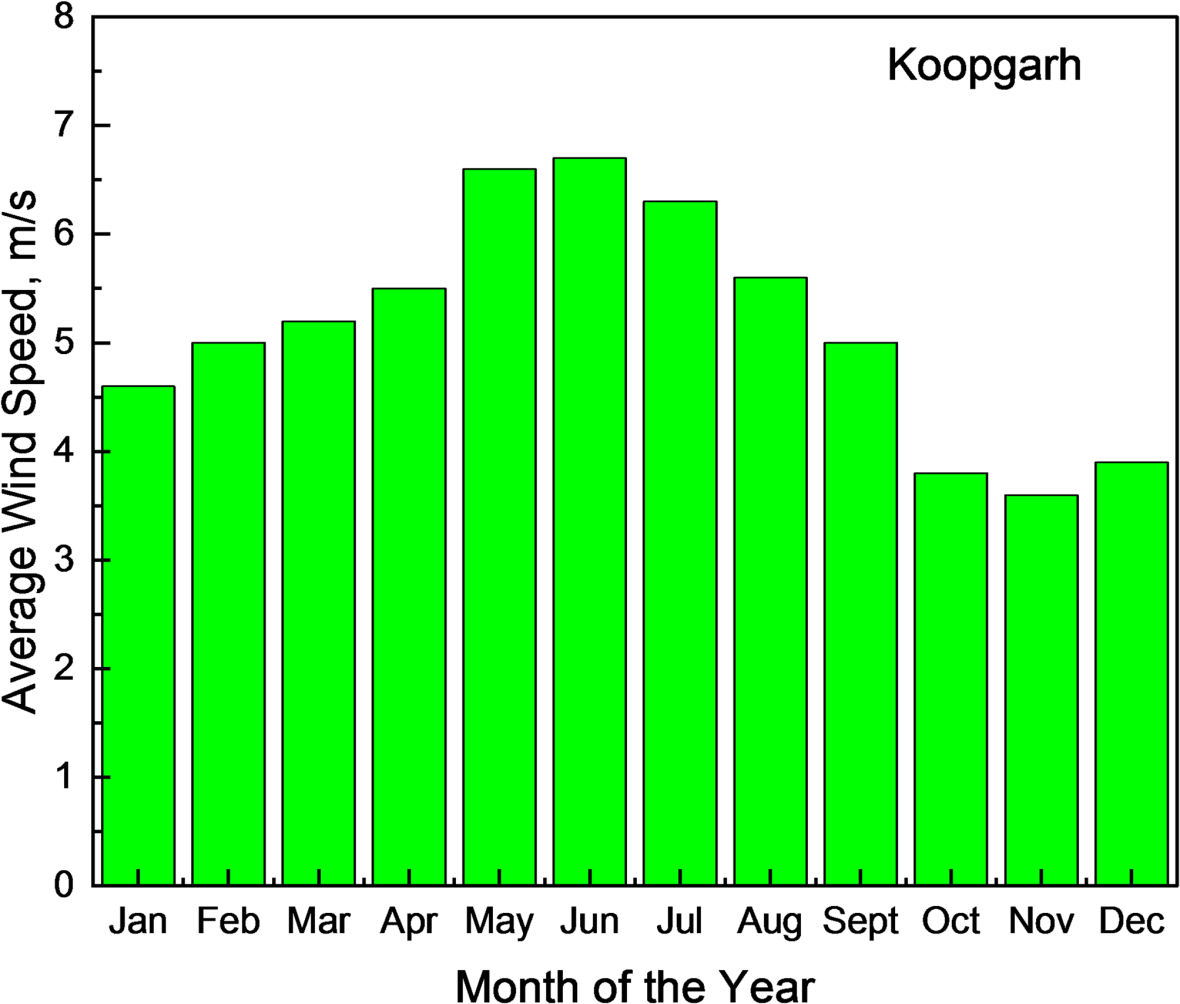
Average Monthly Wind Speed Data at Koopgarh.

The two remote rural sites considered for the analysis were Koopgarh and Kurkheta. Koopgarh, located in the Ashok Nagar district of Madhya Pradesh, is a remote village with minimal access to the central electricity grid of India. The area experiences a subtropical climate, marked by hot and dry summers, and receives abundant solar radiation throughout the year. With a population density of approximately 42 individuals per km^2^, According to data sourced from NASA, the site’s mean annual temperature stands at 25.95 °C, as presented in Fig. 2. The monthly mean daily temperature for the Koopgarh location is shown in Fig. 3. The National Solar Radiation Database further reports that the location receives an average daily solar irradiance of 5.06 kWh/m² annually, as illustrated in Fig. 4. Additionally, wind data obtained from the NASA POWER database indicate that the site has an average yearly wind speed of 4.90 m/s, as shown in Fig. 5.

Table 1 depicts the electrical load distribution among different households in Koopgarh considered for this analysis. These values were used to estimate the total electricity demand for the village under five distinct household load scenarios.

**Table 1:** Average Electrical Load for different households at Koopgarh.

Name of the equipment	Household-1			Household-2			Household-3			Household-4			Household-5		
	A	B	C	A	B	C	A	B	C	A	B	C	A	B	C
Light	4	15	10	2	10	10	2	12	8	4	9	11	3	10	6
Fan	2	90	12	2	90	12	2	90	8	2	90	10	3	90	9
Refrigerator	1	150	24	1	150	24	1	150	24	0	0	0	1	150	24
Water Pump	1	1000	0.5	1	1000	0.5	1	1000	0.75	1	1000	0.75	1	1000	0.5
Devices	1	7	2	1	7	3	1	7	2	1	7	3	1	7	2

A – Quantity; B – Wattage, W; C- Consumption Time in hours.

**Fig. 6:**
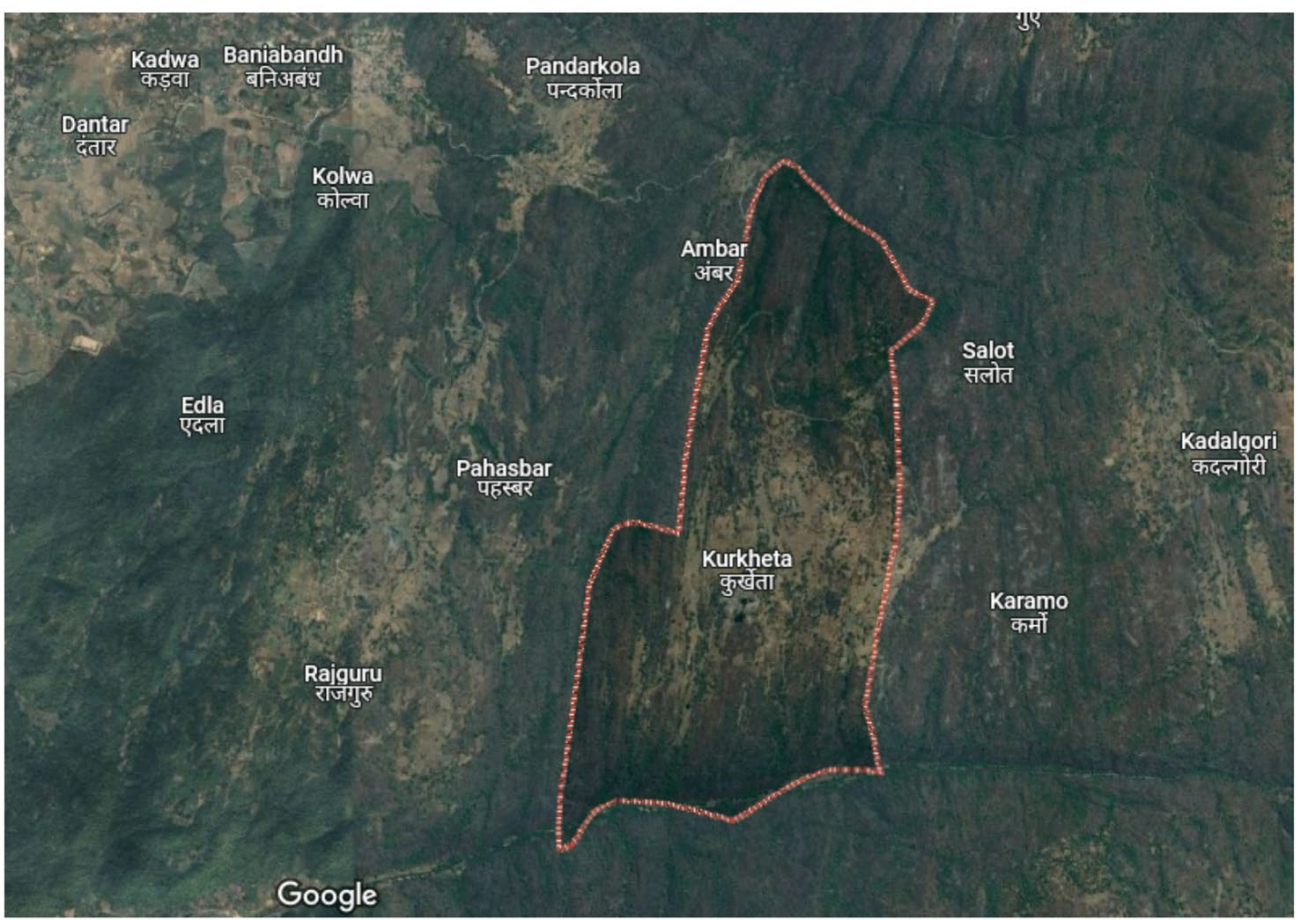
Aerial View Kurkheta village. (Source: Google Maps).

**Fig. 7:**
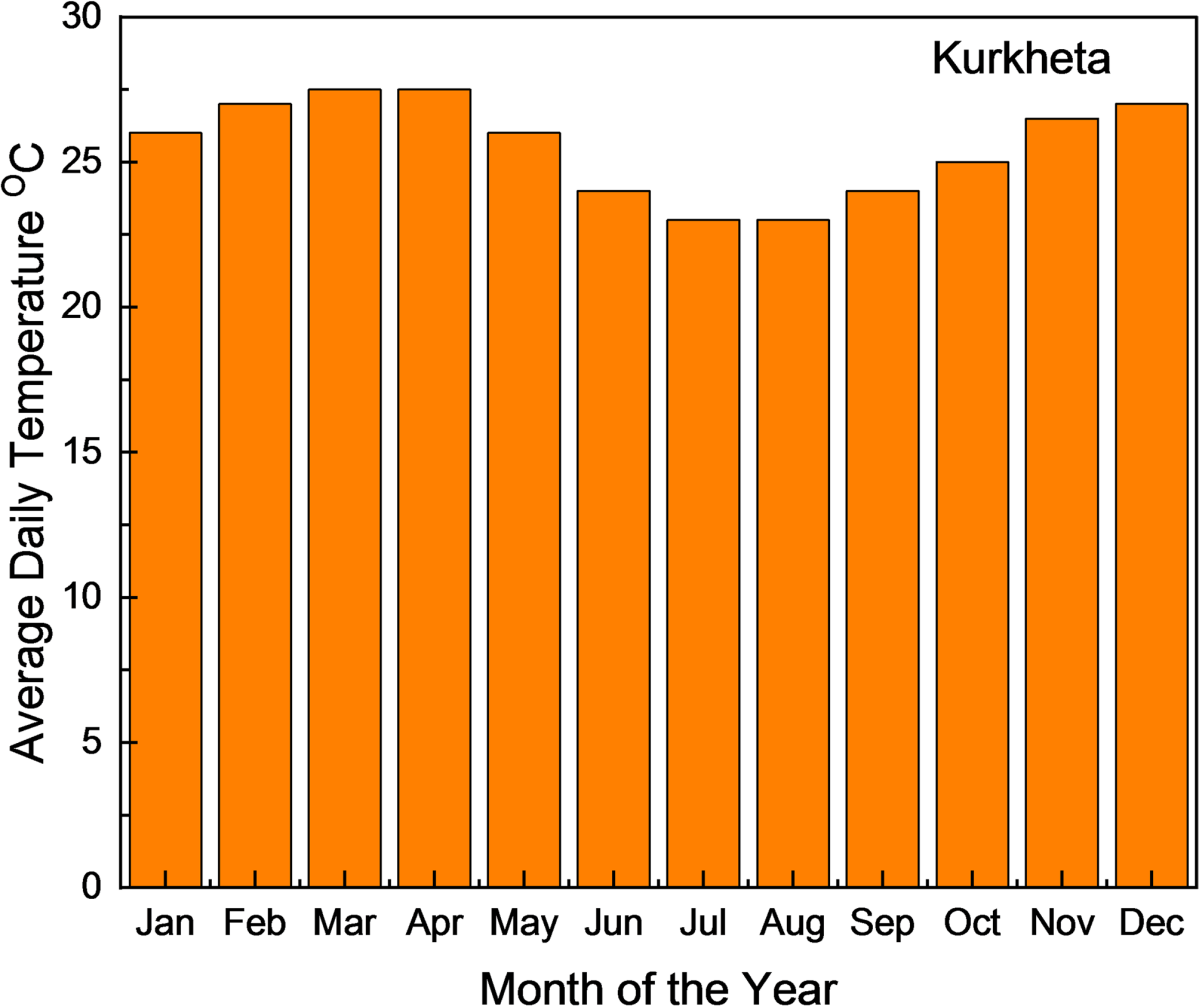
Monthly Average Temperature for Kurkheta location.

Situated between 24°19.0’N and 84°54.4’E, Kurkheta falls under the Shaligram Ram Narayanpur Block in Jharkhand’s Chatra District, as shown in Fig. 6. The region has a humid subtropical climate. According to a 2022 survey, the village comprises 200 households with a total population of approximately 1,200. Although electric poles and wiring are present, the power supply remains unreliable. The NASA POWER database reported an annual average temperature of 25.95 °C for the site, as shown in Fig. 7.

**Fig. 8:**
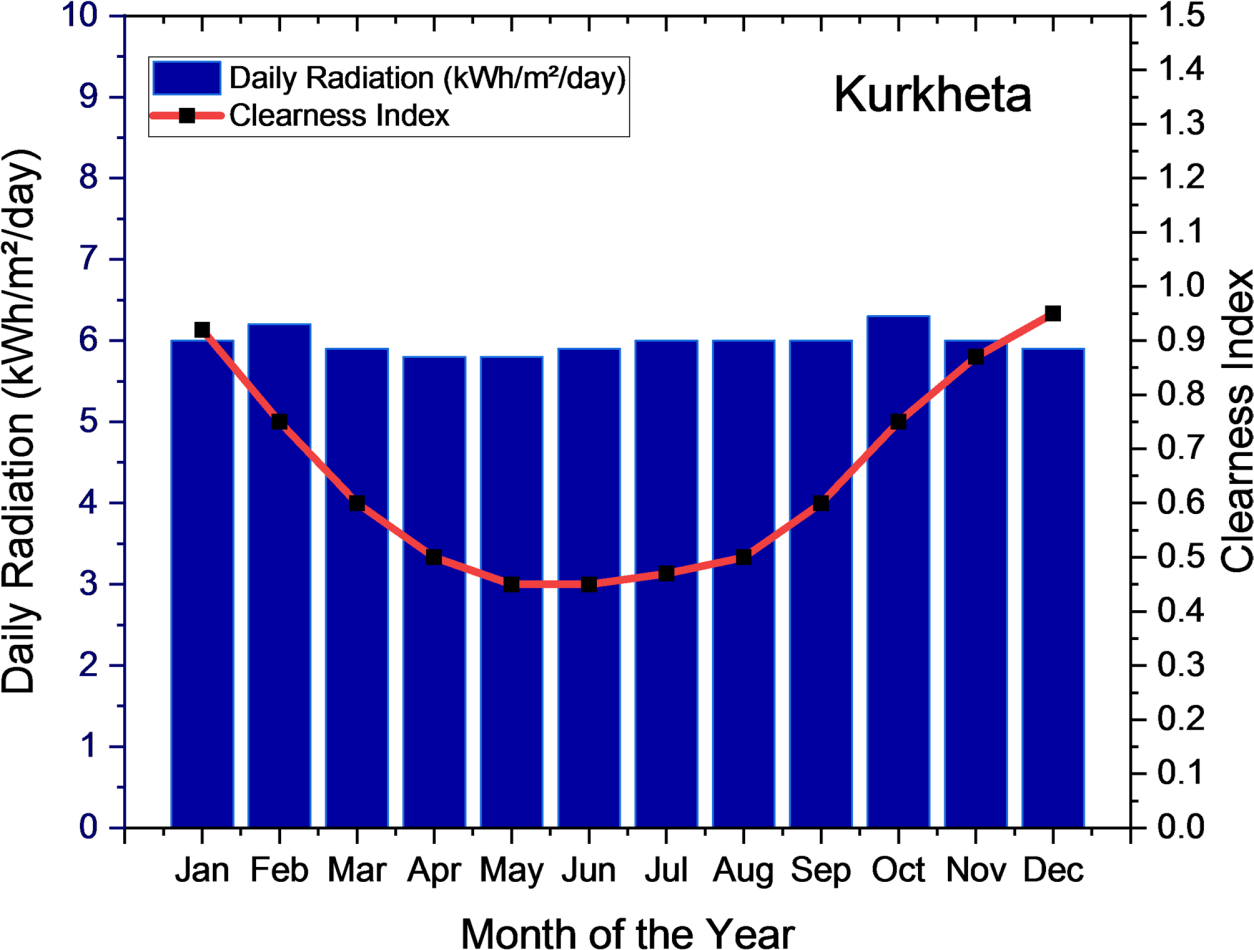
Average Monthly Solar Global Horizontal Irradiance (GHI) Index for Kurkheta.

According to the NSRD, the location receives an average yearly Global Horizontal Irradiance (GHI) of 5.85 kWh/m²/day, along with a clearness index, as shown in Fig. 8. Additionally, the site’s average wind speed is 4.86 m/s per year, based on the NASA POWER data, as illustrated in Fig. 8.

**Fig. 9:**
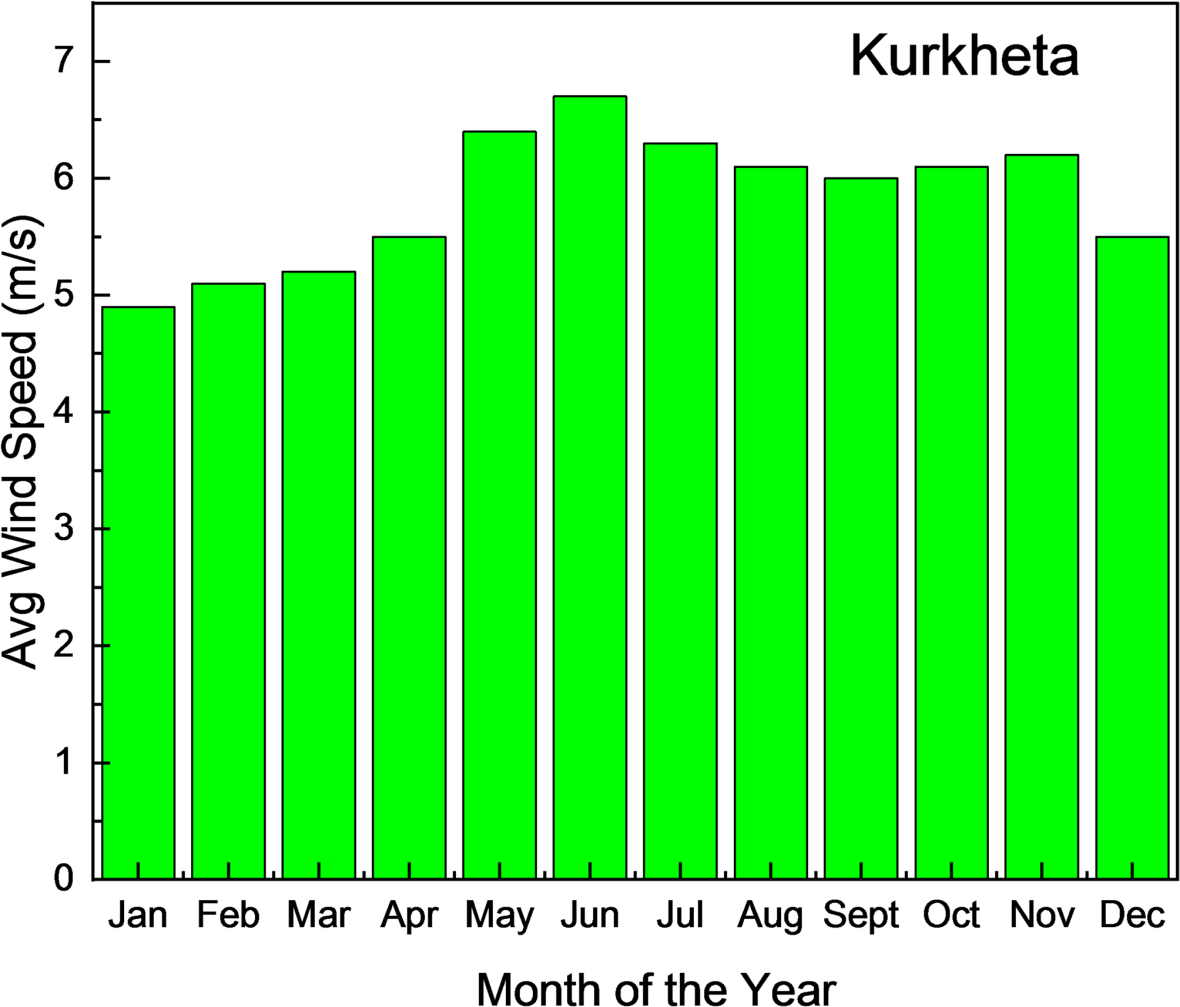
Average Monthly Wind Speed for Kurkheta.

The NASA POWER database reports an annual average temperature of 25.95 °C for the Kurkheta site, and the mean monthly wind speed data are shown in Fig. 9. Table 2 outlines the electrical loads associated with standard households, including the number of appliances, their operational hours, and corresponding energy usage. The average daily energy consumption during summer and winter was 856 kW-h/d, as shown in Table 2. Thus, integrating surplus energy from HRES into the grid can help minimize storage costs and reduce the Net Present Cost (NPC). In the present study, appliance usage was assumed to be relatively consistent across both seasons at the Koopgarh and Kurkheta locations. Calculations were performed for both summer and winter conditions, with the exception of fan usage, which was excluded from the daily load during summer days.

**Table 2:** Average Electrical Load for different households at Kurkheta.

Name of the equipment	Household-1			Household-2			Household-3			Household-4			Household-5		
	A	B	C	A	B	C	A	B	C	A	B	C	A	B	C
Light	3	15	10	2	10	8	3	12	10	3	10	11	2	10	8
Fan	2	90	12	1	90	11	2	90	10	1	90	9	2	90	9
Refrigerator	1	150	24	1	150	24	0	0	0	1	150	24	1	150	24
Water Pump	1	1000	0.5	1	1000	0.75	1	1000	0.5	1	1000	0.5	1	1000	0.75
Devices	1	7	3	1	7	3	1	7	2	1	7	3	1	7	3

A – Quantity; B – Wattage, W; C- Consumption Time in hours.

### Load calculations

The electrical loads presented in Tables 1 and 2 were estimated using the bottom-up household energy assessment method. For each household category, the daily energy consumption of individual appliances was calculated using the product of the appliance quantity, rated power, and average daily operating hours, as expressed by Eq. (1).

1 $$\:{\mathrm{E}}_{\mathrm{appliance}}=\mathrm{N}\times\:\mathrm{P}\times\:\mathrm{T}$$

where $\:\mathrm{N}$ is the number of appliances, $\:\mathrm{P}$ is the rated power (W), and $\:\mathrm{T}$ is the daily operating time (hours). The total daily household energy consumption was calculated by summing the energy use of all household appliances. The village-level load was determined by aggregating the total daily energy demand of all households. Appliance ownership patterns and operating hours were selected based on field survey data, regional rural electrification studies, and typical usage patterns observed in tribal households. Seasonal variations were accounted for by modifying appliance usage durations, particularly for fans that were excluded during the winter months.

**Fig. 10:**
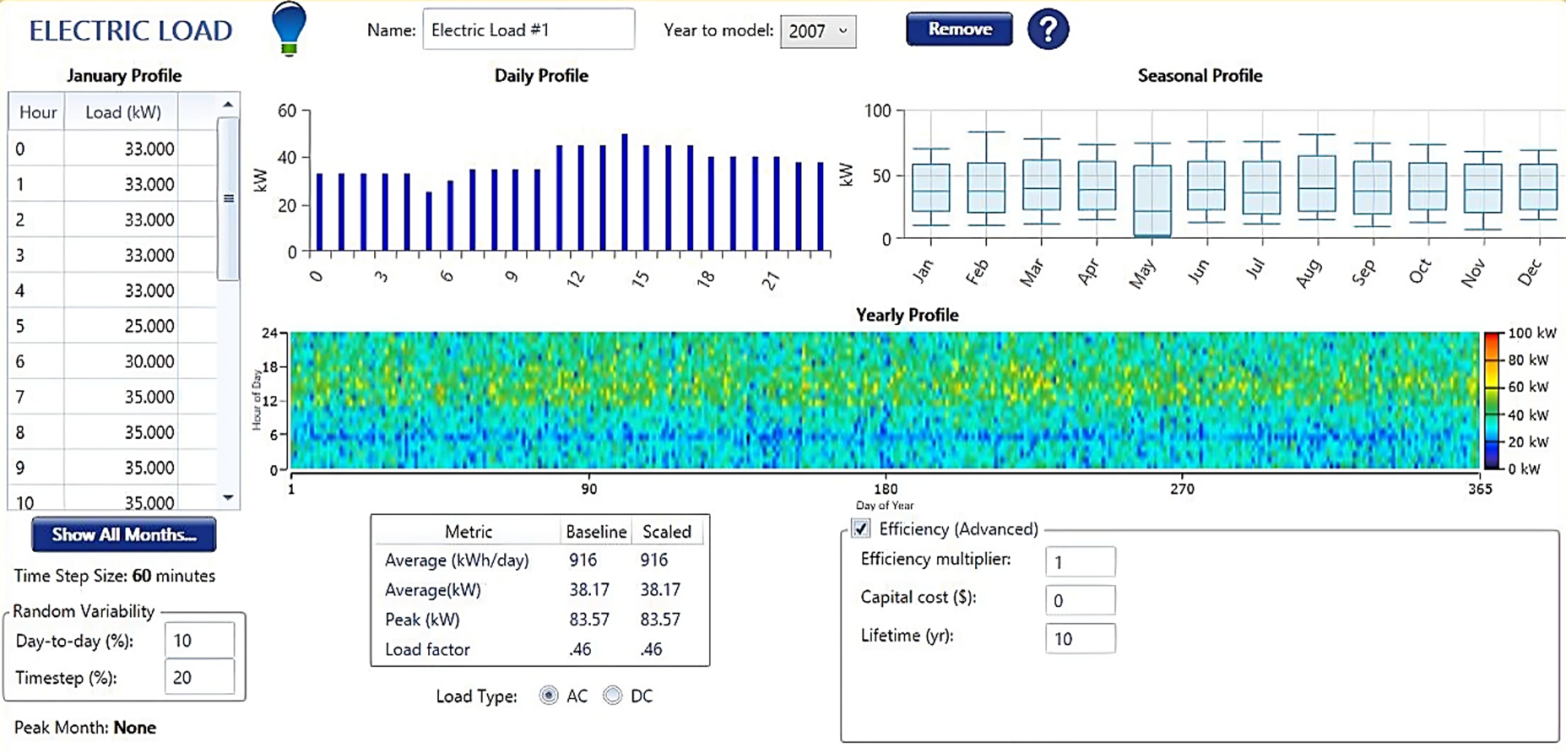
Load Profile of Kurkheta location.

Koopgarh had 91 habitations with a total average daily electrical power consumption of 460.00 kW-h/day. Kurkheta has 200 habitations with a total average daily electrical power consumption of 915.36 kW-h/day. The summer and winter electrical loads vary for the habitations in Koopgarh and are calculated to be 499 and 310.6 kW-h/day, respectively, for the analysis. Similarly, for Kurkheta location, the summer and winter electrical loads for the habitations were calculated as 1167.36 and 663.36 kW-h/day, respectively. Based on the requirements of the Kurkheta location, a load profile was provided to HOMER, as shown in Fig. 10, to conduct the study.

### Specifications of HRES components

**Fig. 11:**
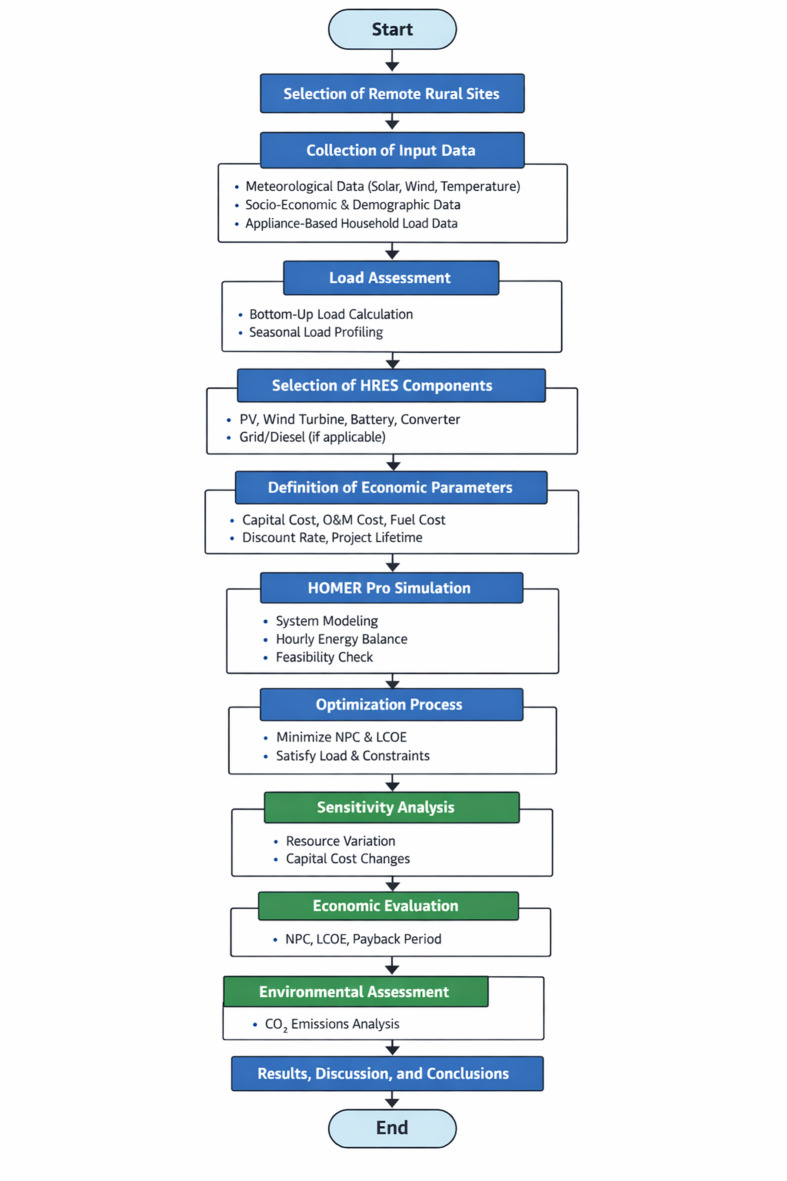
Flowchart of the overall algorithm adopted for HRES modeling, optimization, and techno-economic assessment using HOMER Pro.

The flowchart outlined in Fig. 11 provides a structured methodology for designing a Hybrid Renewable Energy System (HRES) for rural northern India using HOMER Pro. It includes site selection, load assessment, component sizing, economic parameter definition, simulation, and optimization, followed by sensitivity, economic, and environmental analyses to identify the most feasible and cost-effective configuration.

The following components are used in the hybrid renewable energy system analysed here, and their specifications are provided below.

**Solar Panel**.

Solar PV panel specifications are generally provided under standard test conditions (STC). HOMER allows users to run simulations that reflect real-world operating conditions of microgrids. Several factors, such as solar irradiance, temperature, dust, and panel soiling, influence photovoltaic panel performance. Considering an 80% derating factor and a 25-year lifespan for the solar PV panel, the cost economics of the panel are summarized as follows.

Equation (2) provides the output of the PV array.

2 $$\:{P}_{PV}=\:{Y}_{PV}\times\:{f}_{PV}-\:\frac{{IR}_{T}}{{IR}_{T,STC}}\:\left[1+\alpha\:\left({T}_{C}-{T}_{C,STC}\right)\right]$$

Since the effects of temperature on the panel were not considered, the equation becomes:

3 $$\:{P}_{PV}=\:{Y}_{PV}\times\:{f}_{PV}-\:\frac{{IR}_{T}}{{IR}_{T,STC}}$$

For economic analysis, the capital and replacement cost of the panel was taken as 5.78 USD, whereas the cost for operation and maintenance was assumed to be 0.11 USD/year per m^2^ of installation area.

**Wind Turbine**.

The analysis considered a wind turbine with a 3-kWh rated capacity at a hub height of 17 m. The cost economics of wind turbines are as follows. Equations (4) and (5) represent the wind speed at the hub height and the power output of the turbine, respectively.

4 $$\:{V}_{hub\:}=\:{V}_{anem}\times\:\:{\frac{{z}_{hub}}{{z}_{anem}}}^{\alpha\:}$$

Equation (4) represents the

5 $$\:{P}_{WTG}=\:{P}_{WTG,STP}\times\:\frac{\rho\:}{{\rho\:}_{^\circ\:}}$$

For the financial analysis, the capital and replacement costs of the wind turbine were taken as 37.75 USD, whereas the cost for operation and maintenance was assumed to be 0.82 USD/year per kW installation.

### Converter

Many HRES use batteries to store energy, especially when HRES generation is insufficient or unavailable. A key component of such systems is the converter, which can switch between AC and DC. This analysis considered a converter with a 1 kWh rated capacity and 95% efficiency. For the financial analysis, the capital and replacement costs of the converter were taken as 4.36 USD, whereas the cost for operation and maintenance per year was assumed to be nil.

### Battery storage

HOMER provides various storage solutions, including idealized batteries, kinetic batteries, capacitors, flywheels, converters, hydrogen tanks, and electrolyzers. The software factors in the energy throughput of the battery over its lifetime, regardless of the cycle depth, and estimates the battery life. The cost details of the energy storage system of 1-kWh capacity and 3000 kWh throughput was assumed as 4.36 USD, with the cost for operation and maintenance per year as 0.11 USD.

The battery state of charge (SOC) is given by Eq. (6)^39^

6 $$\:\boldsymbol{S}\boldsymbol{O}\boldsymbol{C}\left(\boldsymbol{t}\right)=\boldsymbol{S}\boldsymbol{O}\boldsymbol{C}\left(\boldsymbol{t}-1\right){\int\:}_{\boldsymbol{t}-1}^{\boldsymbol{t}}\frac{{\boldsymbol{P}}_{\boldsymbol{b}}\left(\boldsymbol{t}\right){\boldsymbol{\eta\:}}_{\boldsymbol{b}}}{{\boldsymbol{V}}_{\boldsymbol{b}\boldsymbol{u}\boldsymbol{s}}}$$

where η_b_ is the battery efficiency, V_bus_ is the DC bus voltage, and P_b_(t) is the output power of the battery in KW. The value of P_b_(t) is calculated using Eq. (7) given by^40,41^.

7 $$\:{P}_{b}\left(t\right)=\:\frac{k{Q}_{1}\left(t\right).\mathrm{exp}\left(-k\right)+Q\left(t\right).k.c.(1-\mathrm{exp}\left(-k{\Delta\:}t\right))}{1-\mathrm{exp}\left(-k{\Delta\:}t\right)+c.(\left(k{\Delta\:}t\right)-1+\mathrm{e}\mathrm{x}\mathrm{p}\left(-k{\Delta\:}t)\right)}$$

In the above Eq. (7), Q_1_(t) denotes the amount of energy displayed at the start of the operating interval that is more than the minimum SOC, k refers to the steady-state energy storage constant, and c represents the warehouse space ratio. Q(t) indicates the maximum amount of energy at the start of time, and Δt represents the time interval.

### Diesel

For remote areas, the combination of HRES with a diesel generator can be an effective solution, particularly for smaller energy needs. Diesel generators require more maintenance and fuel, as the cost of diesel fluctuates. This analysis considers inflation rates and sensitivity factors related to diesel prices. The cost of diesel fuel for operating the generator at the Koopgarh and Kurkheta locations was assumed to be USD 1.137 and 1.140, respectively.

## Results and discussion

This study identified two remote locations in India and proposed appropriate HRES configurations to meet the annual household energy needs. The HOMER simulation tool was used to evaluate the energy generation, economic performance, and emissions. Five specific household electrical loads were assumed for each location in the analysis. In addition, the cost of the components, along with the operation and maintenance involved in the installation and operation of the hybrid renewable system, was assumed based on current market conditions. Based on the results obtained from the HRES analysis, a few recommendations and conclusions were derived for each location.

### Criteria for the selection of optimistic system configuration

**Fig. 12:**
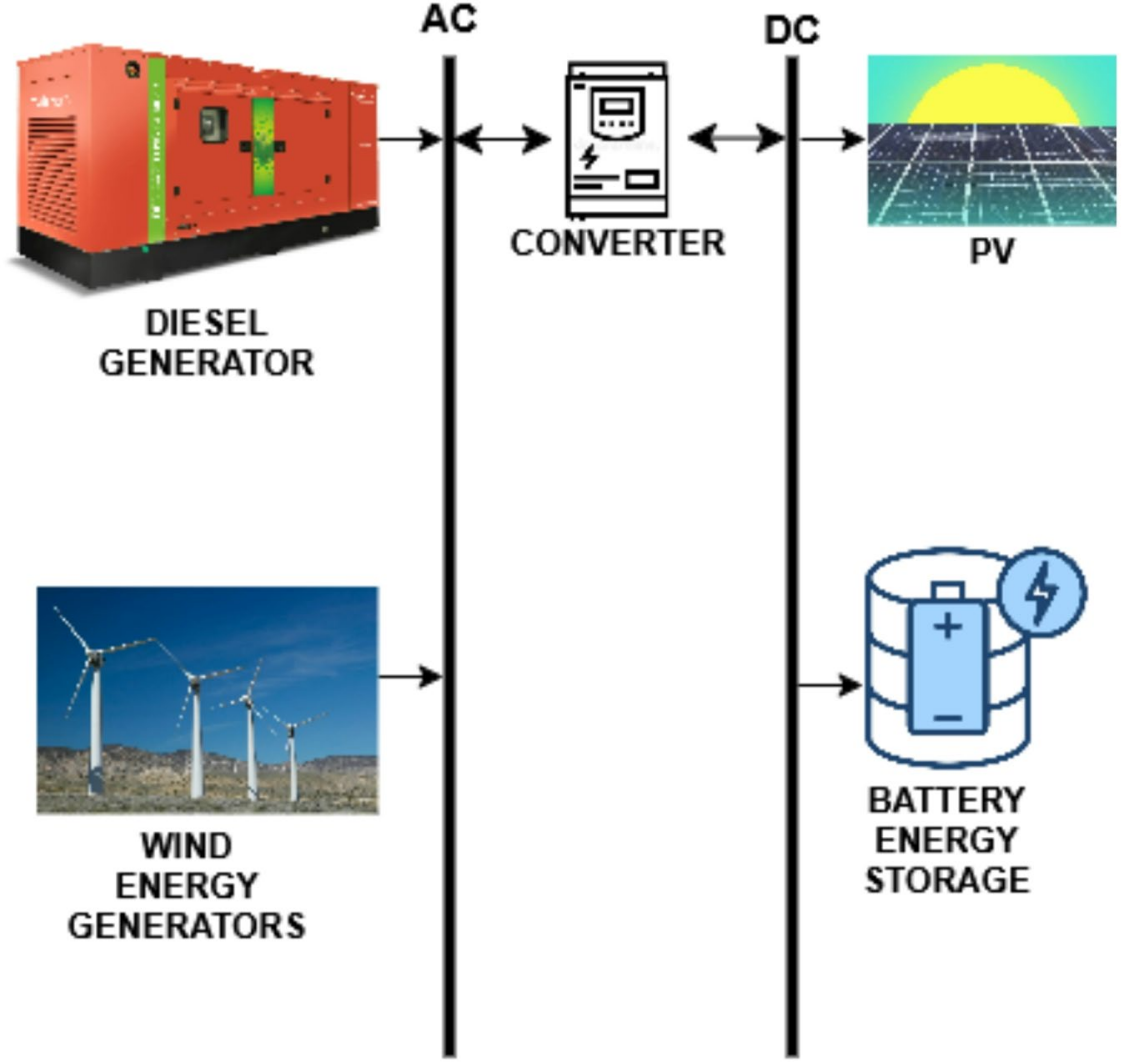
Battery State of Charge for Koopgarh.

HOMER conducts simulations for multiple potential design configurations. Both selected design configurations for the locations incorporated conventional and renewable sources, including diesel generators, solar power, and wind energy, in addition to battery energy storage, as illustrated in Figs. 10 and 12, respectively. The HOMER program selects a design that satisfies the load requirements while adhering to the specified system limitations. Comparisons are made between various combinations of renewable systems for rural electrification and the conventional option of grid extension, using Net Present Cost (NPC) and Carbon Emissions Quantity (CEQ) as key criteria.

The overall net present expenditure of a system is equal to the present worth of all expenses it accumulates throughout its lifespan, minus the present value of all income it generates over its operational lifetime. The costs involved include capital expenditures, replacement costs, operational and maintenance costs, fuel expenses, fines for emissions, and costs of purchasing electricity from the power grid.

Equation (8) provides the calculation of the Net Present Cost (NPC) using the HOMER software.

8 $$\:{C}_{NPC}=\frac{{C}_{ATC}}{CRF(i,{R}_{Proj})}$$

Where,

$$\:{C}_{NPC}$$=Net Present Cost, $.

$$\:{C}_{ATC}$$=Annualized Total Cost, $.

CRF = Capital Recovery Factor.

i= Interest rate, %.

$\:{R}_{proj}$= Lifetime of the Project, years.

For a project lifetime of 20 years and considering the rate of interest as 6%, the value of CRF calculated for the analysis was 0.087.

### Koopgarh

**Fig. 13:**
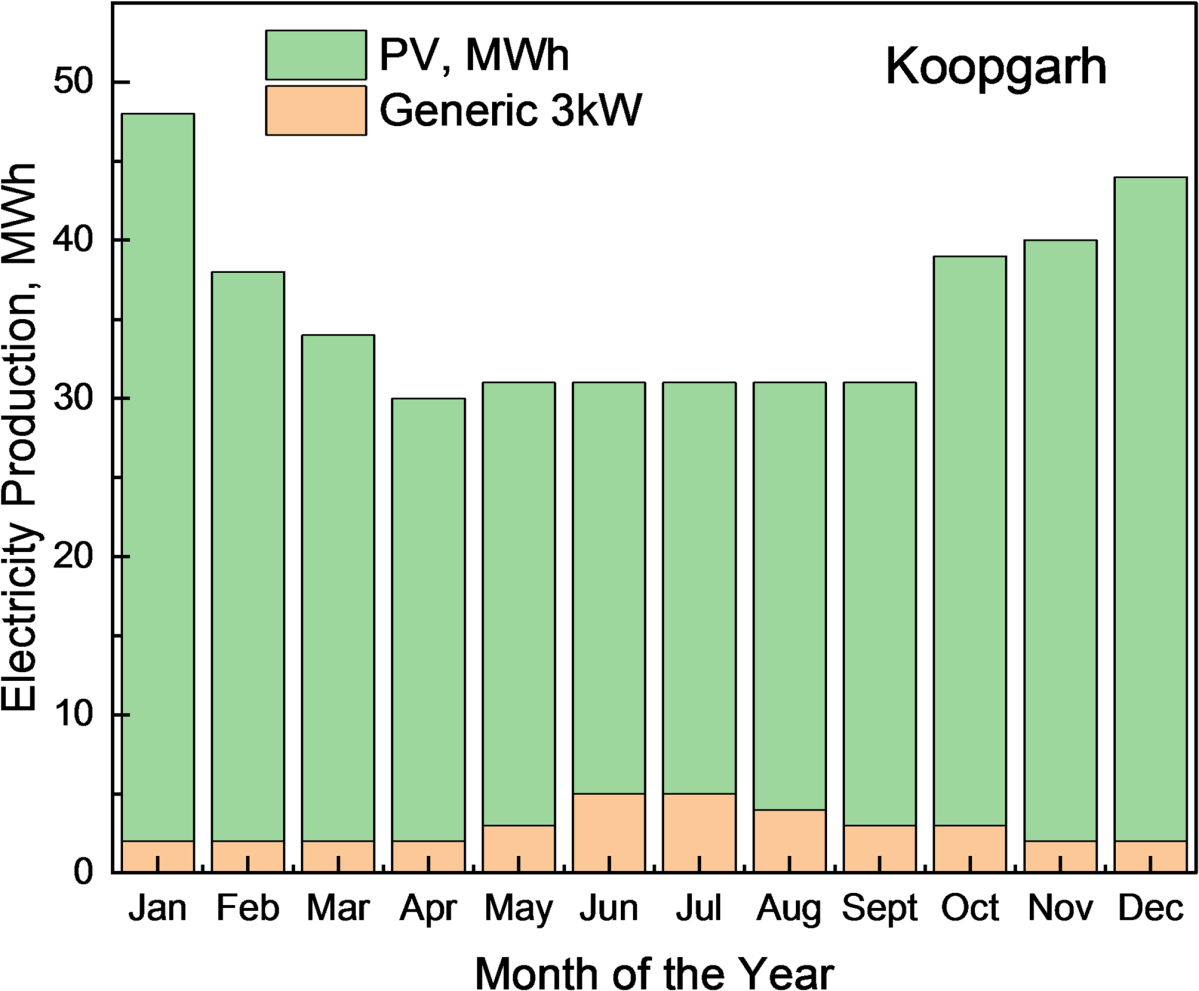
Schematic Diagram of HRES for Koopgarh.

**Fig. 14:**
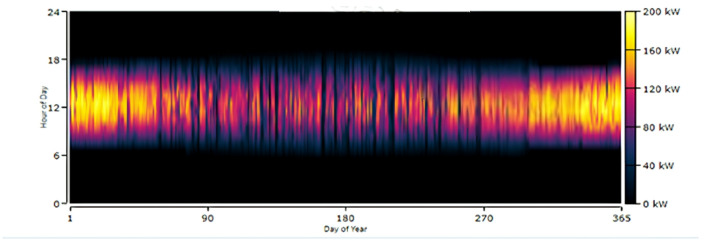
Monthly Electricity Production at Koopgarh for a year from HRES.

**Fig. 15:**
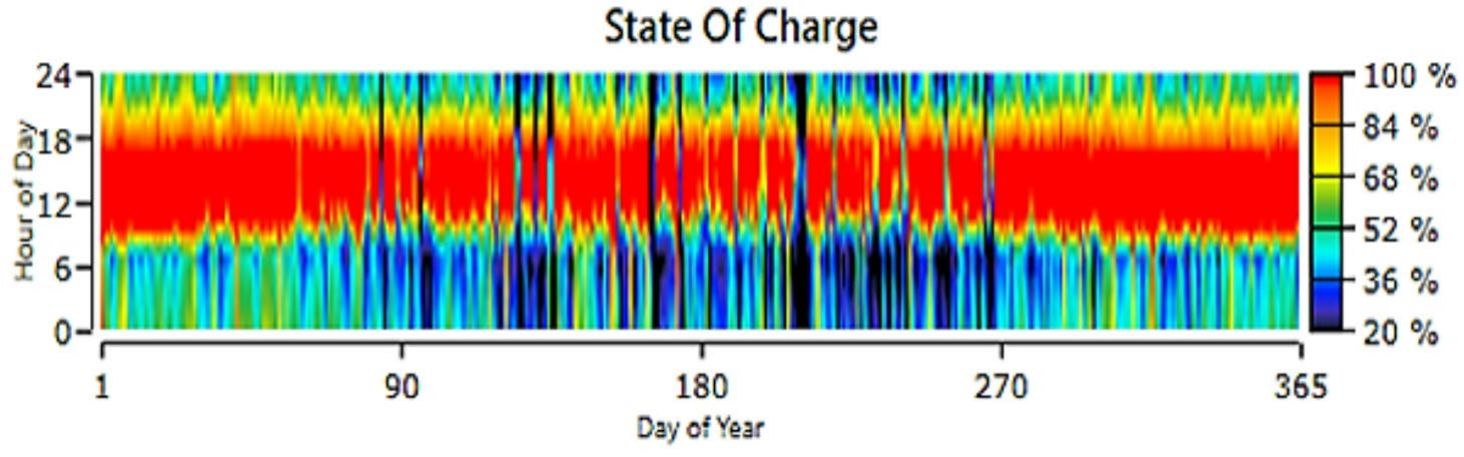
Daily PV Production from HRES at Koopgarh.

The selected HRES configuration for Koopgarh is shown in Fig. 13, which includes a 168-kW flat-plate PV, system converter, and 18-kW wind turbine. The wind turbine contributed 7.29% of the total energy requirement, whereas the PV system generated 92.7%. Figure 14 depicts the combined annual power production from the HRES, in which the solar panel generates between 80 and 200 kWh between 06:00 and 18:00 IST. Figure 15 shows the hourly PV power generation throughout the year for the location. Figure 12 illustrates the state of charge, demonstrating that the storage fills any energy gaps. From Fig. 14, it can be observed that during the months of May to August, the combined power production from wind and solar is moderate. Furthermore, wind energy power production is higher from June to August than in other months of the year. In addition, from October to February, the combined power production from the HRES is higher, whereas the contribution of Solar PV power is greater than that of wind energy. The hourly power generation pattern for a year from solar PV and battery state of charge is depicted in Figs. 15 and 12, respectively. It is noteworthy to observe from Fig. 12 that during the day between 06:00 and 18:00 h IST, the battery state of charge is consistently 100% throughout the year, proving the ability of HRES to meet the energy demands.

### Economics

**Fig. 16:**
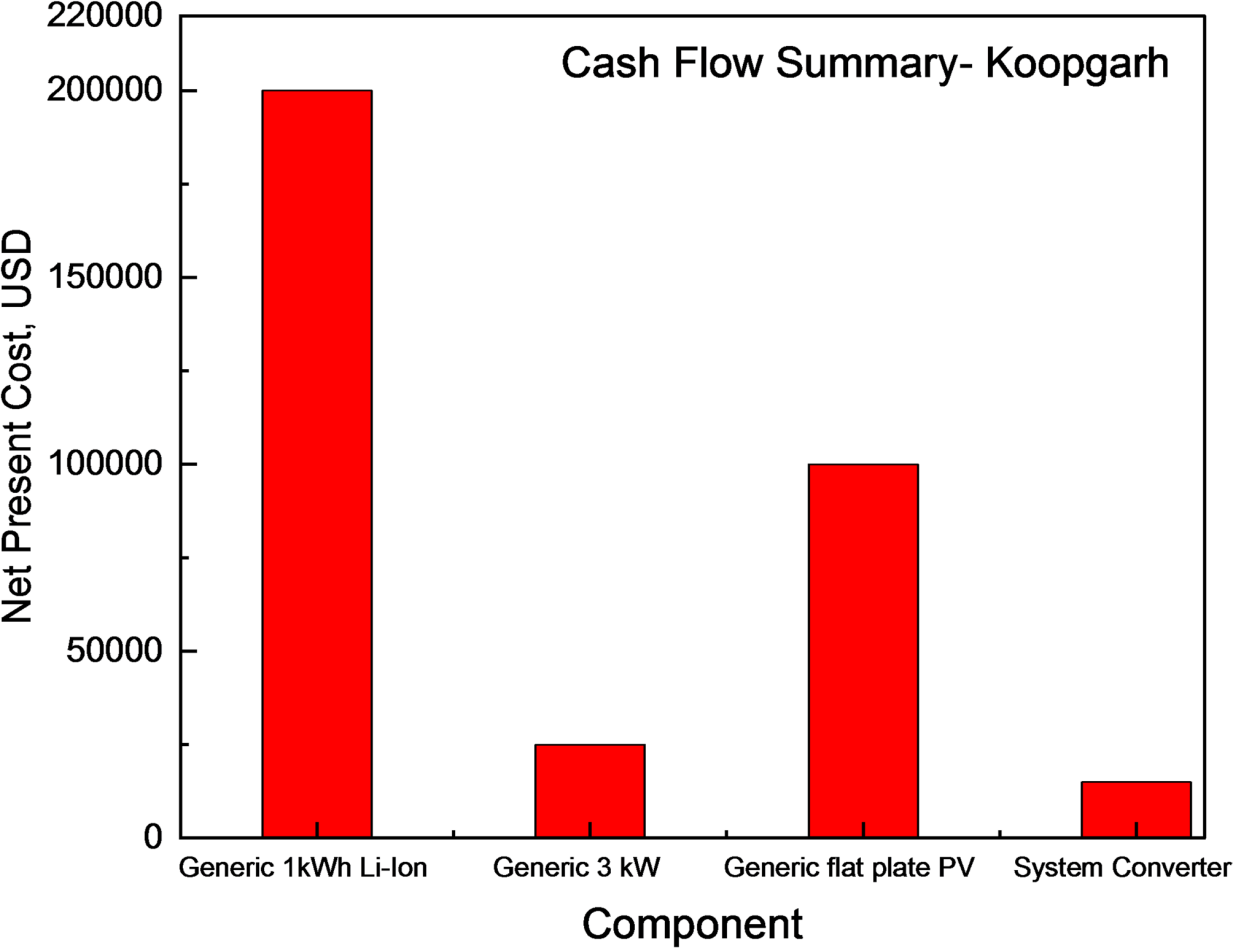
Cash flow Summary of different off-grid systems for Koopgarh location.

The configuration suggested by HOMER’s setup assistant generated 194 kWh from solar energy and 72 kWh from the diesel generator, which was the primary source. The NPC and LCOE of the system were USD 427,749.88 and USD 0.28, respectively. In comparison, the optimized model had an NPC of USD 338,927.61 and an LCOE of USD 0.19, reflecting a lower operating cost of USD 17,798.75. An optimized cash summary for the different off-grid systems at the Koopgarh location is presented in Fig. 16.

### Emissions

In HOMER simulations, emissions are calculated by multiplying the emission factor (EF) by the total fuel consumed. The goal of these simulations was to reduce grid dependency and meet electricity demands using HRES. This approach significantly lowers emissions compared to traditional systems. By adopting HRES, reliance on grid electricity is reduced, leading to lower emissions and contributing to a cleaner, more sustainable energy solution. The recommended schematic achieves zero-net emissions.

### Kurkheta

**Fig. 17:**
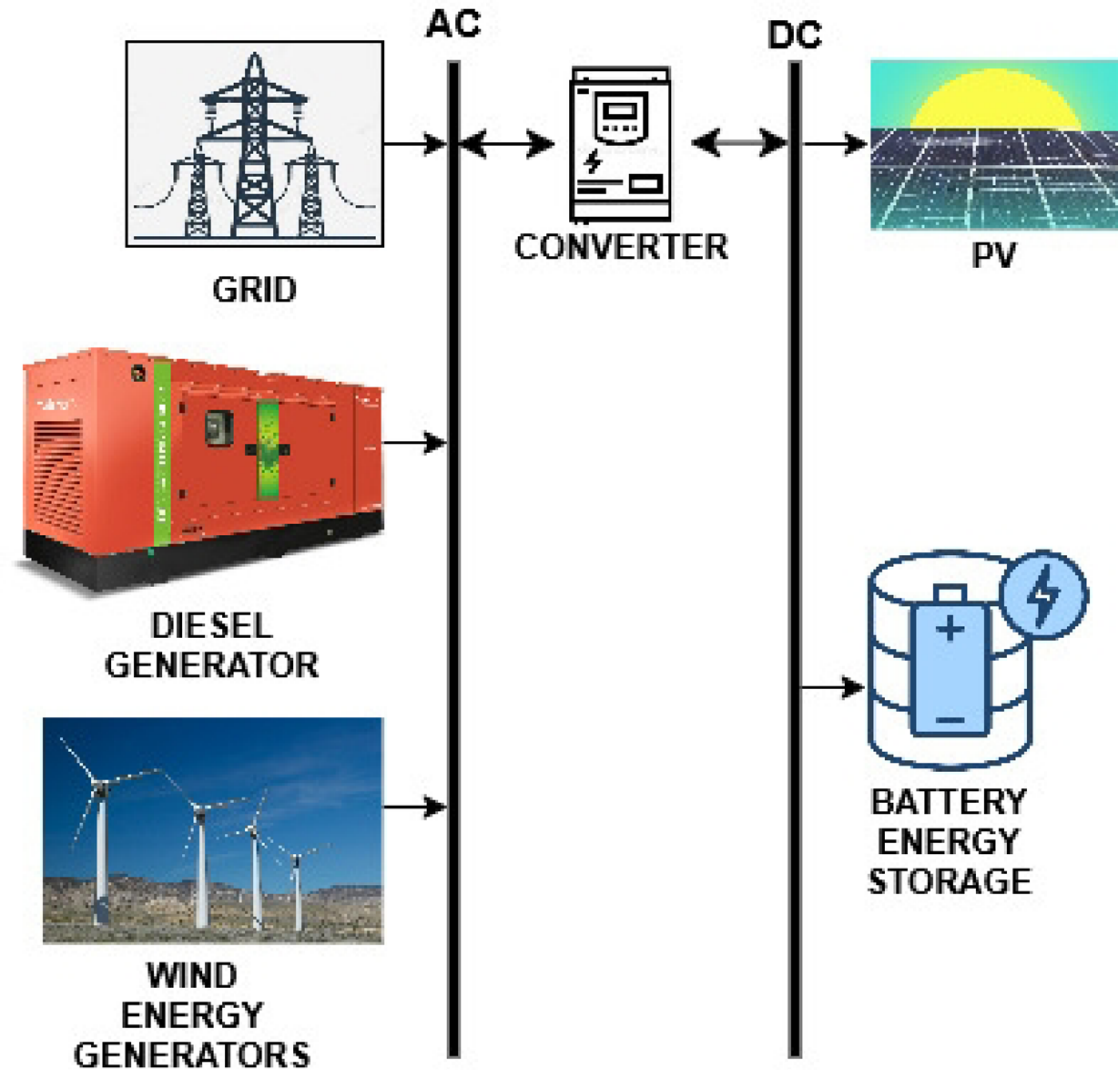
Schematic diagram of HRES for Kurkheta.

**Fig. 18:**
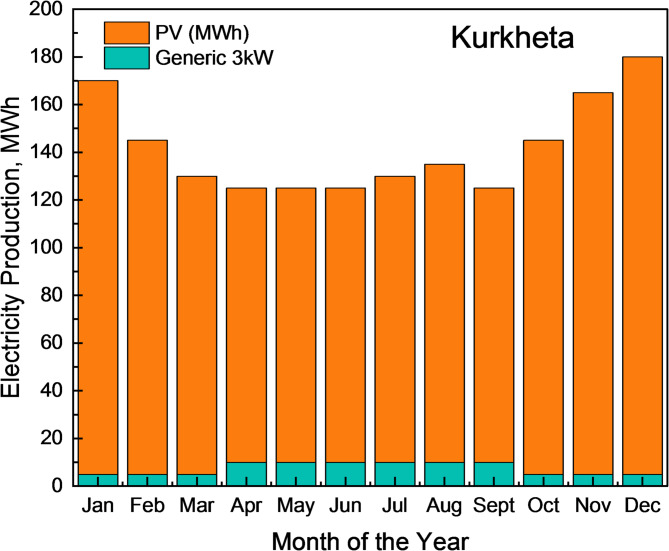
Monthly Electricity Production at Kurkheta for a year from HRES.

**Fig. 19:**
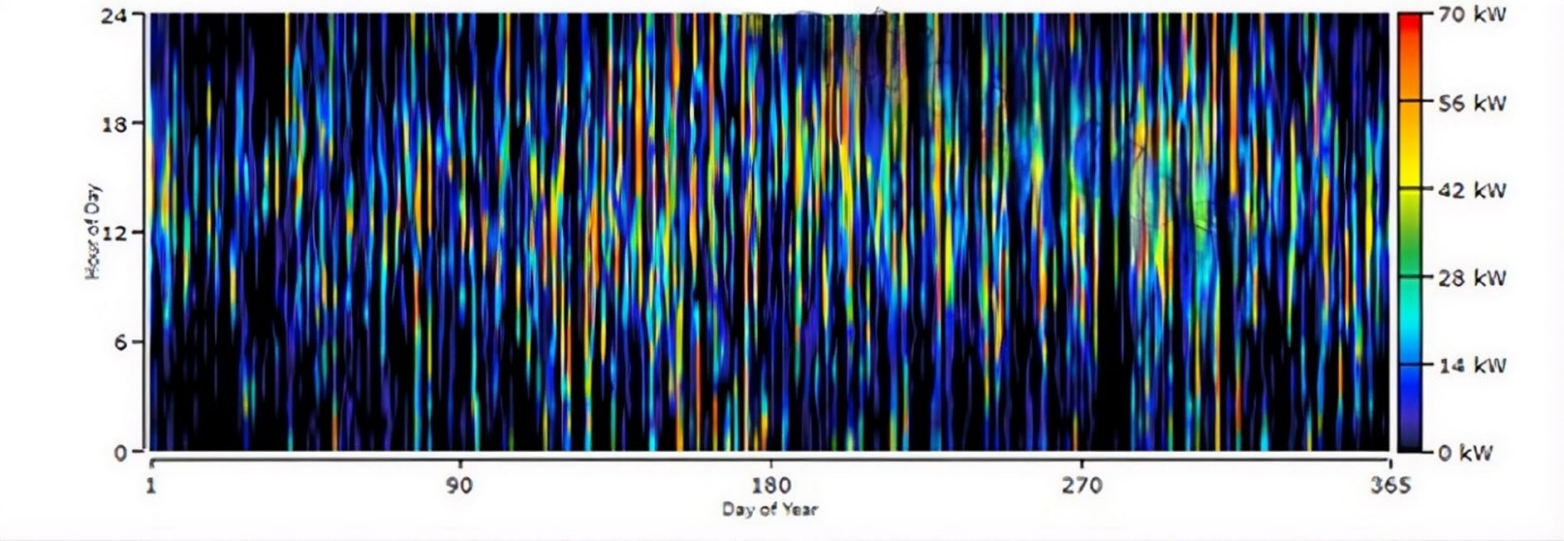
Daily PV Production at Kurkheta from HRES.

**Fig. 20:**
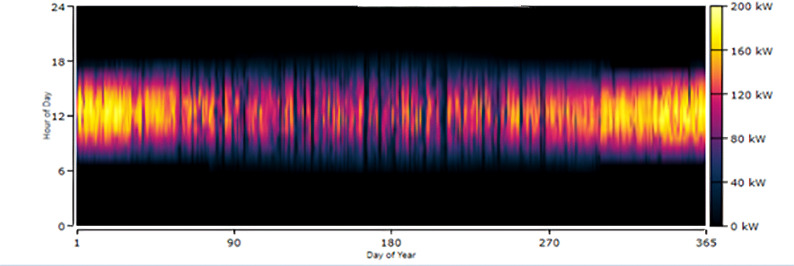
Annual Wind Turbine Output at Kurkheta site from HRES.

The HOMER simulation initially proposed a PV-generator-storage-grid-converter system, which generated 150 kWh from the generator and 1818 kWh from solar power. However, this configuration would require a diesel generator, which produces greenhouse gases and requires regular maintenance. The recommended alternative is a PV-Turbine-Storage-Converter system, shown in Fig. 17, which generates 1,394,879 kWh annually from PV panels and 88,071 kWh from turbines, as shown in Figs. 18, 19 and 20.

This configuration eliminates the need for a diesel generator and significantly reduces greenhouse gas emissions. From Fig. 18, it can be observed that from April to September, the combined power production from wind and solar is moderate. Furthermore, wind energy power production is higher from April to September than in other months of the year. In addition, from October to March, the combined power production from the HRES is higher, whereas the contribution of Solar PV power is greater than that of wind energy. The hourly power generation patterns for a year from solar PV and wind energy are depicted in Figs. 19 and 20, respectively.

**Fig. 21:**
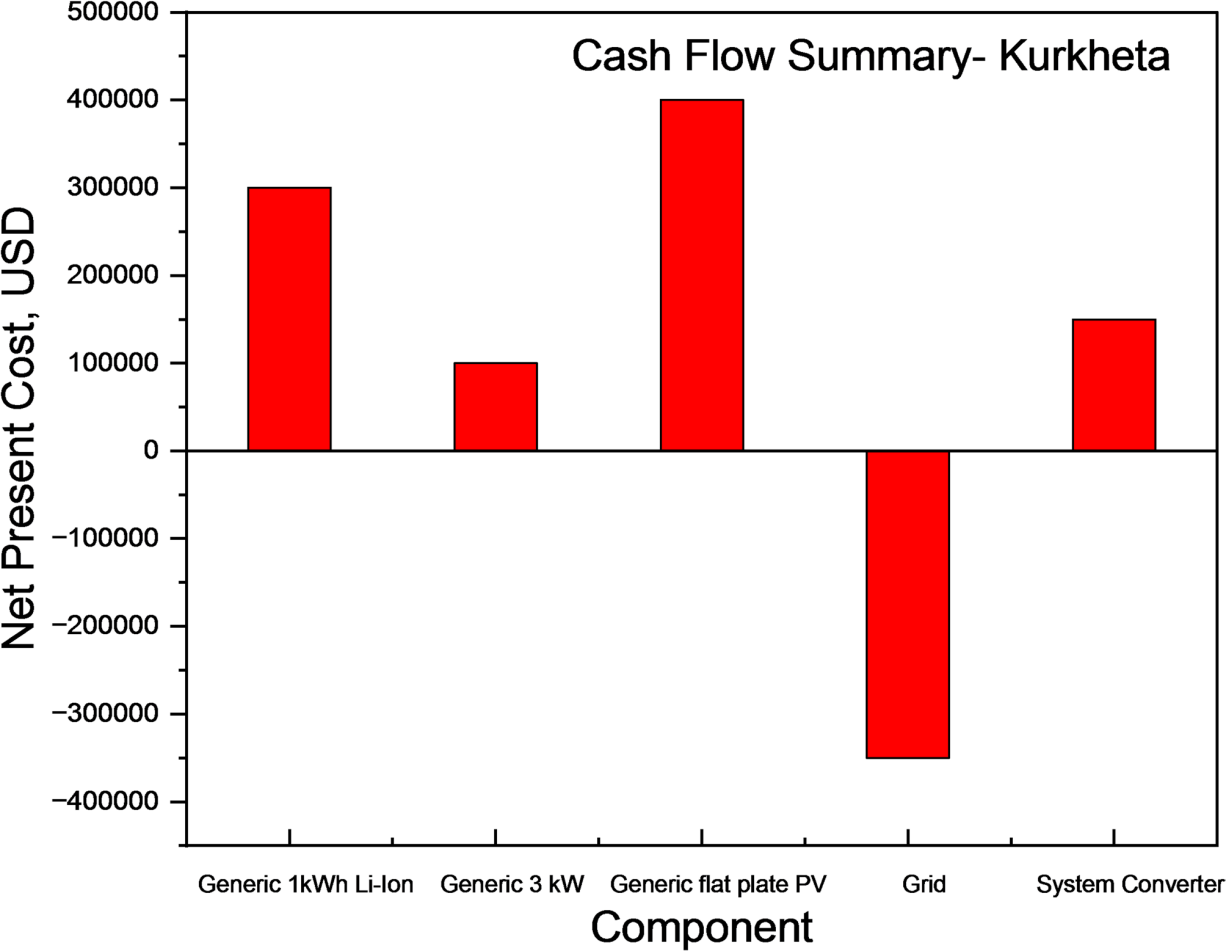
Cash flow Summary of different off-grid systems for Kurkheta.

The initial PV-generator-storage-grid-converter setup had an NPC of USD 623,220.67 and an LCOE of USD 0.024. Owing to the grid connection, excess energy can be fed back into the grid, reducing the NPC and LCOE of the optimized system to USD 486,902.99 and USD 0.033, respectively. The proposed PV-Turbine-Storage-Converter system provides better NPC, as shown in Fig. 21, compared to the other configurations.

### Emissions

The recommended configuration was designed to achieve zero emissions, similar to the Koopgarh schematic. This system is intended to meet the local population’s energy needs without relying on the grid. Although some emissions may occur if electricity is drawn from the grid, the overall system maintains zero net emissions. The total emissions can be calculated by multiplying the EF by net grid purchases. The PV-Turbine-Storage-Converter system is more environmentally friendly and cost-effective, eliminating the need for a diesel generator, reducing greenhouse gas emissions, and sustaining zero-net emissions.

### Validation of results and sensitivity analysis

Based on previous results reported in the literature, the current results validate the proposed system in terms of NPC and CO2 emissions, as shown in Table 3.

#### Sensitivity analysis

The results of the objective function for the optimized HRES may vary with time owing to the impact of changes in NPC values and a shortage of capital^42^. Sensitivity analysis deals with changes in energy resource availability over time and economic fluctuations, which directly influence techno-economic analysis.

**Fig. 22:**
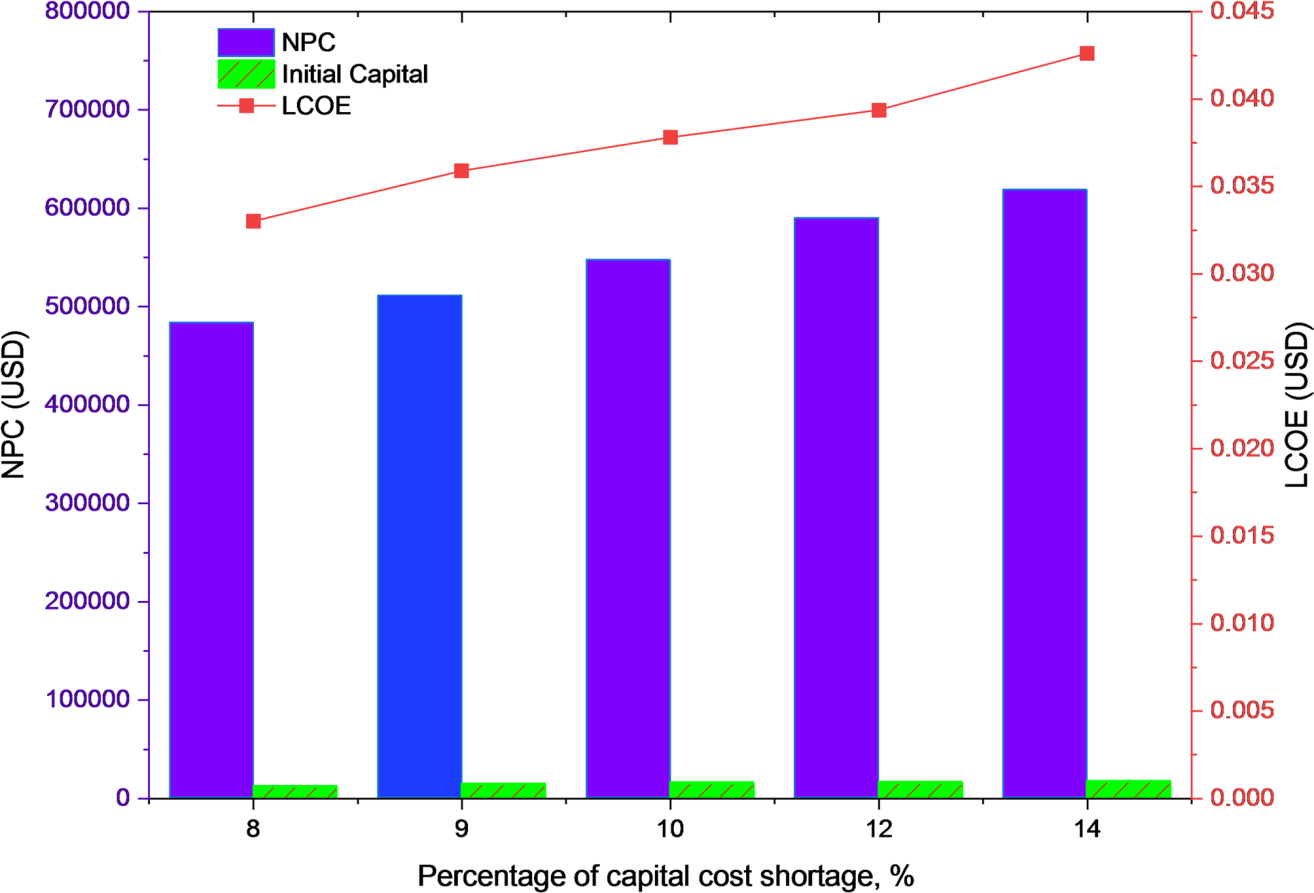
Impact of capital cost shortage on the NPC and LCOE for Kurkheta location.

Figure 22 highlights the results of the sensitivity analysis performed for the proposed HRES at Kurkheta. As observed in Fig. 22, as the percentage of capital cost shortage increases, the LCOE increases, exhibiting a linear trend due to higher NPC values. This analysis suggests a compromise between viable economic benefits and the variability of renewable energy resources over time. Hence, with the proposed system and using the HOMER optimization simulation, a balance between cost-effectiveness, dependability, and environmental sustainability, facilitating wider use in off-grid situations, can be achieved. In summary, the proposed HRES solution can satisfy the present and future energy demands of the locations considered in this study^43^.

**Table 3:** Validation of proposed system results.

Ref.	Location	System Structure	CO_2_ (kg/year)	NPC ($)
^29^	Kilkadayam, India	Wind+Solar+Hydro+Battery	1,15,394	1,62,987
^30^	Punjab, India	DG + PV+Biomass	NA	90,256
^31^	Muhavoor, India	PV+Wind + DG+Battery	5.1 × 10^6^	25,500,000
	Present Study	Solar + DG+Battery+Grid+Wind	0	4,32,218
		Solar + DG+Battery+Wind	0	

#### Payback period analysis

The simple payback period (SPP) of the proposed HRES was calculated to assess the time required to recover the initial capital investment through annual cost savings compared to conventional grid/diesel-based power supply as shown in Table 4. The payback period was determined using Eq. (9).

9 $$\:\mathrm{Payback\:Period\:(years)}=\frac{{\mathrm{C}}_{\mathrm{initial}}}{{\mathrm{C}}_{\mathrm{annual,ref}}-{\mathrm{C}}_{\mathrm{annual,HRES}}}$$

where $\:{\mathrm{C}}_{\mathrm{initial}}$is the initial capital cost of the HRES, $\:{\mathrm{C}}_{\mathrm{annual,ref}}$is the annual cost of electricity supply from the reference system (grid/diesel), and $\:{\mathrm{C}}_{\mathrm{annual,HRES}}$is the annual operating cost of the proposed HRES. Based on HOMER Pro simulation results, the optimized HRES configuration exhibits a reasonable payback period, demonstrating its economic feasibility for long-term rural electrification. Although the initial capital investment is higher than conventional systems, the reduced fuel consumption, lower operational costs, and zero-emission benefits result in attractive lifecycle economics.

**Table 4:** Payback Period calculation for the HRES.

Location	Optimized HRES Configuration	Initial Capital Cost (USD)	Annual Cost (USD/year)	Annual operating Cost HRES (USD/year)	Annual Savings (USD/year)	Simple Payback Period (years)
Koopgarh	PV–WT–Battery–Converter	245,000	39,500	17,799	21,701	11.3
Kurkheta	PV–WT–Battery–Grid–Converter	312,000	58,600	24,950	33,650	9.3

#### Practical applications and deployment potential

The optimized HRES configurations for Koopgarh and Kurkheta demonstrate clear applicability beyond household electrification needs. With average daily energy availability of 460 and 915.36 kWh/day, respectively, the systems are capable of supporting essential community services, including primary healthcare centers, vaccine refrigeration, drinking water pumping, digital classrooms, and rural communication infrastructure. In Kurkheta, surplus energy exported to the grid further enables productive rural enterprises, such as agro-processing units, small-scale food preservation, and micro-industrial activities.

From an energy policy perspective, the obtained LCOE values (USD 0.19 for Koopgarh and USD 0.033 for Kurkheta) indicate that decentralized PV–WT–Battery systems can serve as cost-competitive alternatives to conventional grid extension in geographically isolated terrains. Particularly in Koopgarh, where grid expansion requires substantial transmission infrastructure, the proposed HRES provides a financially viable, decentralized solution. The proposed configurations directly contribute to Sustainable Development Goal 7 (Affordable and Clean Energy) by ensuring reliable electricity access with high renewable energy penetration and near-zero direct operational emissions (DOEs). Unlike diesel-based rural electrification models, the recommended systems eliminate dependence on fuel transport and reduce exposure to fossil fuel price volatility, thereby strengthening long-term energy security.

In terms of SDG 13 (Climate Action), the transition from diesel-supported systems to renewable-dominant HRES significantly reduces greenhouse gas emissions. The elimination of diesel generators in the optimized configuration minimizes carbon emissions, improves local air quality, and reduces public health risks associated with particulate emissions in confined rural environments. Furthermore, the availability of reliable electricity enables productive load integration, indirectly supporting SDG 8 (Decent Work and Economic Growth) by facilitating rural entrepreneurship, value-added agricultural processing and micro-enterprises. The electrification of community infrastructure, such as schools and healthcare centers, also aligns with SDG 3 (Good Health and Well-being) and SDG 4 (Quality Education), as energy access improves healthcare delivery and digital learning opportunities.

Importantly, the modular and scalable architecture of the proposed HRES ensures adaptability to future load growth, thereby supporting long-term sustainable rural development pathways. Thus, the present techno-economic results provide not only an optimized engineering solution but also a replicable framework aligned with national and global sustainability objectives for decentralized rural electrification.

## Limitations of the Study

Despite providing valuable techno-economic insights into hybrid renewable energy systems (HRES) for remote rural electrification, this study is subject to certain limitations. First, the analysis relied on secondary meteorological data obtained from databases such as NASA POWER and NSRDB. While these datasets are widely used and reliable for preliminary feasibility assessments, they may not fully capture the microclimatic variations at the local level. The incorporation of long-term ground-measured meteorological data could further enhance the accuracy of the simulation results. Furthermore, electrical load profiles were developed based on representative household appliance usage patterns and assumed seasonal variations. Although this bottom-up approach reflects typical rural consumption behavior, actual demand may vary owing to socio-economic changes, electrification of productive loads, and population growth. Future studies should incorporate real-time or smart meter-based load data to better capture dynamic demand variations.

A techno-economic evaluation was conducted using HOMER Pro under predefined economic assumptions, including component costs, discount rate, fuel prices, and system lifetime. Variations in market prices, policy incentives, and financing mechanisms may influence the economic performance of the proposed HRES configurations. Finally, the present study focuses on PV–wind–battery-based configurations and does not explicitly consider emerging technologies such as hydrogen energy storage, agrivoltaics, or floating photovoltaic systems owing to data and site-specific constraints. The inclusion of these technologies, along with life cycle assessment (LCA) and embodied carbon analysis, can provide a more comprehensive sustainability evaluation in future studies.

## Conclusions and future scope of work

The proposed Hybrid Renewable Energy Systems (HRES), which integrate various energy sources, effectively meet sustainable energy demands. Simulations for two remote areas with on-grid challenges yielded optimal configurations to address their current and future energy needs while reducing generation costs and emissions. The key findings of this study are as follows.

- The optimal HRES configuration for both Koopgarh and Kurkheta meets the annual energy demands and ensures continuous, sustainable energy flow throughout the system’s life cycle. In the sensitivity scenarios, both regions eliminated the need for fuel-based components, leading to a significantly reduced NPC and LCOE.
- NPC and LCOE values for the optimized HRES designs at Koopgarh and Kurkheta were determined. Owing to the need for transmission and distribution infrastructure at Koopgarh, its NPC and LCOE are higher than those at Kurkheta. While the HRES at Koopgarh cannot be connected to the existing grid, Kurkheta’s configuration can, although the current grid infrastructure does not meet the minimum energy demand of the population. The proposed HRES in Kurkheta generates excess energy, which can be sold, resulting in an NPC of USD 486,902.99 and LCOE of USD 0.033.
- The LCOE of the proposed HRES at both locations is lower than the cost of conventional grid power in the surrounding areas.

By incorporating advanced technologies, the efficiency of individual components and overall system performance can be enhanced, further lowering the LCOE. Additionally, emissions can be reduced by adopting a circular economy approach at the component and system levels. For future work, to enhance the system reliability, ground-based meteorological data can be used as input to the simulation, which is one of the limitations of this study. Limited HRES options were considered in the present analysis, which can be extended to the latest technological sustainable practices in terms of resource availability for further reductions in the system NPC. The life cycle analysis (LCA) of these HRES systems can be conducted by determining the embodied carbon emissions following a basic carbon accounting methodology. This can provide better insights into adopting these optimized simulation results for specific regional energy demands.

### Skilled manpower and scalability considerations

One of the key challenges in implementing HRES in remote rural and tribal regions is the limited availability of skilled manpower for system installation, operation, and maintenance. Advanced components, such as inverters, battery energy storage systems, and power electronics, require trained personnel to ensure reliable performance and minimize downtime. In many rural areas, the absence of locally trained technicians can lead to increased system outages, higher maintenance costs, and a dependence on external service providers.

To address this challenge, capacity-building initiatives such as localized training programmes, community-based technical workshops, and partnerships with regional technical institutes are essential. Developing a locally skilled workforce not only enhances system reliability but also promotes employment generation and community ownership, thereby improving the long-term sustainability of HRES installations in the Philippines.

Furthermore, the modular and scalable nature of HRES allows for phased expansion to meet the increasing energy demands associated with population growth, electrification of productive loads, and improved living standards. By integrating additional renewable generation units and energy storage modules, HRES can be expanded without major infrastructural modifications, making them well suited for rural electrification programs. Such scalability, combined with local capacity development, enables the wider replication of the proposed HRES framework across remote regions with similar geographic and socioeconomic conditions.

### Land use, community acceptance, and emerging technologies

Land availability and competing land-use priorities present significant challenges for the large-scale deployment of renewable energy systems in rural and tribal regions. In many communities, land is primarily utilized for agriculture, forestry, and habitation, making it difficult to allocate dedicated areas for solar or wind installations without affecting livelihoods or causing social resistance to the project. Therefore, community acceptance is a crucial factor in the successful implementation of HRES projects. To address these challenges, modern and innovative technologies, such as agrivoltaics and floating photovoltaics (flotovoltaics), offer promising alternatives. Agrivoltaic systems enable the simultaneous use of land for agricultural production and solar power generation, thereby optimizing land productivity and providing additional income streams to rural households. Similarly, floating photovoltaic systems installed on water bodies, such as ponds, reservoirs, or irrigation tanks, can generate electricity without occupying valuable land while reducing water evaporation.

Incorporating such technologies into HRES frameworks can significantly reduce land-use conflicts, enhance community participation, and improve the social acceptance of renewable energy projects. Although the present study focuses on ground-mounted PV–wind–battery systems owing to site-specific constraints and data availability, future expansions of the proposed HRES can integrate agrivoltaics or flotovoltaics where suitable, further improving sustainability and replicability across diverse rural settings.

## Data Availability

The datasets used and/or analyzed during the current study are available from the corresponding author upon reasonable request.

## Abbreviations

DSM: Demand Side Management
GHG: Greenhouse Gas
HRES: Hybrid Renewable Energy System
LCOE: Levelized Cost of Energy
NPC: Net Present Cost
PSO: Particle Swarm Optimization
PV: Photovoltaics
WES: Wind Energy Systems
